# Potentiation of Epithelial Innate Host Responses by Intercellular Communication

**DOI:** 10.1371/journal.ppat.1001194

**Published:** 2010-11-18

**Authors:** Tamas Dolowschiak, Cécilia Chassin, Sanae Ben Mkaddem, Thilo M. Fuchs, Siegfried Weiss, Alain Vandewalle, Mathias W. Hornef

**Affiliations:** 1 Institute of Medical Microbiology and Hospital Epidemiology, Hannover Medical School, Hannover, Germany; 2 INSERM U773, Centre de Recherche Biomédicale Bichat-Beaujon, Paris, France; 3 Université Paris 7-Denis Diderot, site Bichat, Paris, France; 4 ZIEL, Department of Microbiology, Technical University Munich, Freising, Germany; 5 Molecular Immunology, Helmholtz Centre for Infection Research, Braunschweig, Germany; Institut Pasteur, France

## Abstract

The epithelium efficiently attracts immune cells upon infection despite the low number of pathogenic microbes and moderate levels of secreted chemokines per cell. Here we examined whether horizontal intercellular communication between cells may contribute to a coordinated response of the epithelium. *Listeria monocytogenes* infection, transfection, and microinjection of individual cells within a polarized intestinal epithelial cell layer were performed and activation was determined at the single cell level by fluorescence microscopy and flow cytometry. Surprisingly, chemokine production after *L. monocytogenes* infection was primarily observed in non-infected epithelial cells despite invasion-dependent cell activation. Whereas horizontal communication was independent of gap junction formation, cytokine secretion, ion fluxes, or nitric oxide synthesis, NADPH oxidase (Nox) 4-dependent oxygen radical formation was required and sufficient to induce indirect epithelial cell activation. This is the first report to describe epithelial cell-cell communication in response to innate immune activation. Epithelial communication facilitates a coordinated infectious host defence at the very early stage of microbial infection.

## Author Summary

All body surfaces are covered by a single layer of epithelial cells. Epithelial cells form a physical barrier to separate the underlying sterile tissue from the environment. In addition, epithelial cells actively sense bacterial and viral infection. The recognition of pathogenic microorganisms results in cell stimulation and the secretion of soluble mediators that attract professional immune cells to the site of infection. This first line host defence works very efficiently despite the often low number of pathogens and the limited amount of mediators secreted per epithelial cell. We therefore investigated whether infection of one individual epithelial cell would result in activation of other, non-infected cells within a confluent epithelial monolayer resulting in a more substantial host response. Indeed, using the model of the gut pathogen *Listeria monocytogenes* and monitoring infection and epithelial activation at a single cell level, we can clearly show that the epithelial response is mainly mediated by non-infected cells. Also, we identify oxygen radicals as potential mediators to facilitate horizontal epithelial communication upon immune stimulation. Our results thus provide a novel concept of a coordinated epithelial host response upon microbial infection facilitated by horizontal epithelial communication.

## Introduction

Intestinal epithelial cells line the enteric mucosal surface and provide a physical barrier to maintain the integrity of this vulnerable body surface and prevent invasive infection by luminal microorganisms. Like professional immune cells, intestinal epithelial cells express receptors of the innate immune system such as Toll-like receptors (TLR) or nuclear oligomerization domain (NOD)-like receptors (NLR) [1], [2]. Recognition of microbial structures leads to epithelial production of antimicrobial effector molecules and proinflammatory chemoattractive mediators. Thus, it facilitates an active role in the initiation of the mucosal host response [3], [4], [5]. The recruitment of professional immune cells to the site of infection occurs within hours and provides a highly efficient dynamic mechanism of the epithelial host defence. It remains unclear, however, how low number of pathogenic microorganisms as well as the limited spectrum and only moderate amount of chemokine secretion per epithelial cell facilitates stimulation of an effective host defence. We therefore hypothesized that a horizontal intercellular communication between intestinal epithelial cells might help to induce a coordinated epithelial response towards infectious challenge and thereby to amplify the epithelial innate host defence.

*Listeria monocytogenes* is an important human pathogen that causes meningitis, sepsis, and abortion in susceptible individuals. It is acquired with food such as unpasteurized milk and cheese and enters the body following penetration through the intestinal epithelial barrier. The microbial pathogenesis and the bacteria-host cell interaction of this facultative intracellular bacterium has been studied for many years [6]. *L. monocytogenes* induces its own internalization and subsequently lyses the endosomal membrane of its host cell by the secretion of listeriolysin O (LLO) and phospholipases, thus gaining access to the cytosolic space. Here, *Listeria* upregulates polar expression of ActA that recruits and polymerizes host actin filaments resulting in propulsive locomotion. Together with LLO and the phospholipases this allows to enter neighbouring cells and to spread within the epithelial cell layer. Importantly, recognition of *Listeria* by the epithelial innate immune system only occurs after internalization and lysis of the endosomal membrane through cytosolic innate immune receptors [7], [8], [9], [10]. Since infection of individual cells can be traced using reporter gene technology, *L. monocytogenes* provides an excellent model to study cellular responses in respect to immune recognition at the single cell level.

In the present study, we analyzed innate immune recognition and epithelial responses at the single cell level using the model of *Listeria* infection of polarized intestinal epithelial cells in addition to transfection and microinjection. We present the surprising finding that non-infected epithelial cells were the main source of chemokine secretion in response to bacterial challenge. We identify oxygen radical species produced by NADPH oxidase (Nox) 4 in response to cytosolic bacteria to facilitate horizontal intercellular communication and chemokine production by non-infected cells. These results provide the first experimental evidence for a yet unknown mechanism of intercellular communication between epithelial cells in response to innate immune stimulation and thus significantly broaden our understanding of mucosal innate host defence.

## Results

### Invasion-dependent recognition but indirect epithelial cell activation after *Listeria* infection

**Figure 1:**
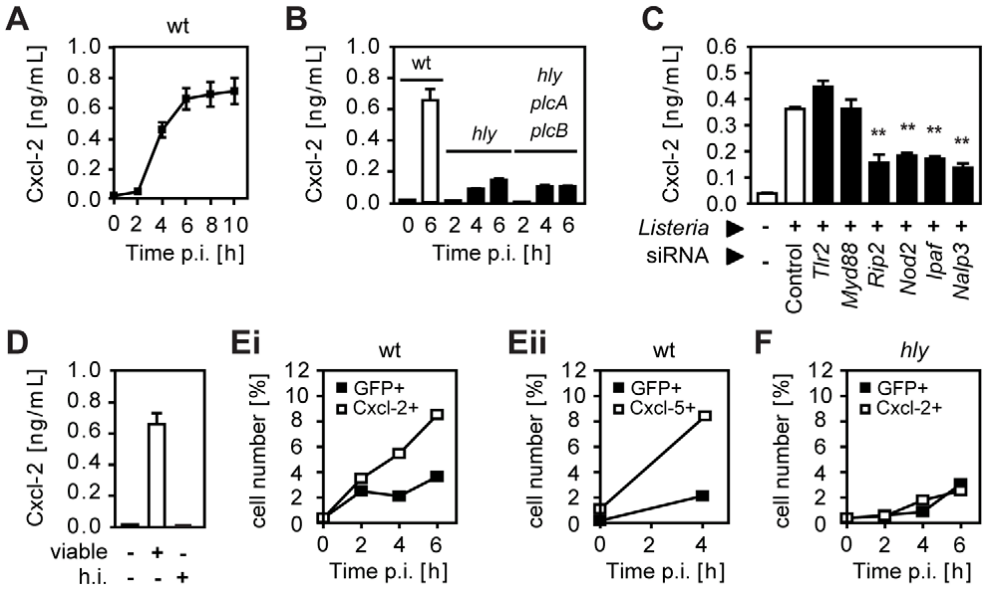
*Listeria*-induced activation of intestinal epithelial cells is largely dependent on invasion and endosomal lysis. (**A**) m-IC_cl2_ cells were infected with wild-type (wt) *Listeria monocytogenes*. Cxcl-2 was determined after the indicated time in cell culture supernatant by ELISA. (**B**) m-IC_cl2_ cells were infected with wt, *hly* mutant or *hly*/*plcA*/*plcB* triple mutant *L. monocytogenes*. Cxcl-2 was determined after the indicated time in cell culture supernatant by ELISA. (**C**) m-IC_cl2_ cells were treated with small interfering RNA (siRNA) control or siRNA *Tlr2*, *MyD88*, *Rip2*, *Nod2*, *Ipaf* or *Nalp3* and infected with *actA* mutant *L. monocytogenes*. Cxcl-2 was determined 4 h after infection in cell culture supernatant by ELISA. **, p<0.01. (**D**) m-IC_cl2_ cells were infected with viable or heat inactivated (h.i.) wt *L. monocytogenes*. Cxcl-2 was determined 6 h after infection in cell culture supernatant by ELISA. (**E**) m-IC_cl2_ cells were infected with wt *L. monocytogenes* expressing green fluorescence protein (GFP) under control of the *actA* promoter (P*_actA_*-*gfp*). The number of GFP^+^ (*Listeria*-infected, black square) or immunolabelled (**Ei**) Cxcl-2^+^ or (**Eii**) Cxcl-5^+^ (white square) cells was determined after the indicated time by flow cytometry. (**F**) m-IC_cl2_ cells were infected with *hly* mutant P*_actA_*-*gfp L. monocytogenes*. The number of GFP^+^ (*Listeria*-infected, black square) or immunolabelled Cxcl-2^+^ (white square) cells was determined after the indicated time by flow cytometry. All experiments were performed at a multiplicity of infection of 100∶1. Results are representative for three independent experiments and are presented as mean ± SD (ELISA) or show one representative experiment.

Infection of a confluent monolayer of intestinal epithelial m-IC_cl2_ cells with wild-type (wt) *L. monocytogenes* induced rapid cellular activation illustrated by secretion of the proinflammatory chemokine Cxcl-2 (Fig. 1A). Strong epithelial activation was only observed using wt *Listeria* able to reach the cytosolic space (Fig. 1B) facilitating recognition by cytoplasmic innate immune receptor molecules (Fig. 1C) [7], [8], [9], [10]. Bacterial mutants unable to lyse the endosomal membrane such as isogenic *hly* or *hly*/*plcA*/*plcB* triple mutants as well as heat inactivated bacteria exhibited a significantly reduced or even absent epithelial activation (Fig. 1B and D). Of note, lack of *hly* or *hly*, *plcA*, and *plcB* expression did not affect bacterial invasion or intracellular viability (Fig. S1A). Endosomal lysis-dependent stimulation of *L. monocytogenes* infected epithelial cells was also observed using flow cytometry. A time-dependent increase of the number of Cxcl-2***^+^*** and Cxcl-5***^+^*** epithelial cells was detected after infection with wt *Listeria* (Fig. 1E). In contrast, a strongly reduced number of epithelial cells stained positive for Cxcl-2 after infection with *hly* mutant *Listeria* (Fig. 1F). In accordance with the published literature, internalization-dependent activation was observed in epithelial cells, but not in macrophages (Fig. S1B). These results suggested that activation of epithelial cells occurred primarily in directly *Listeria*-infected cells.

**Figure 2:**
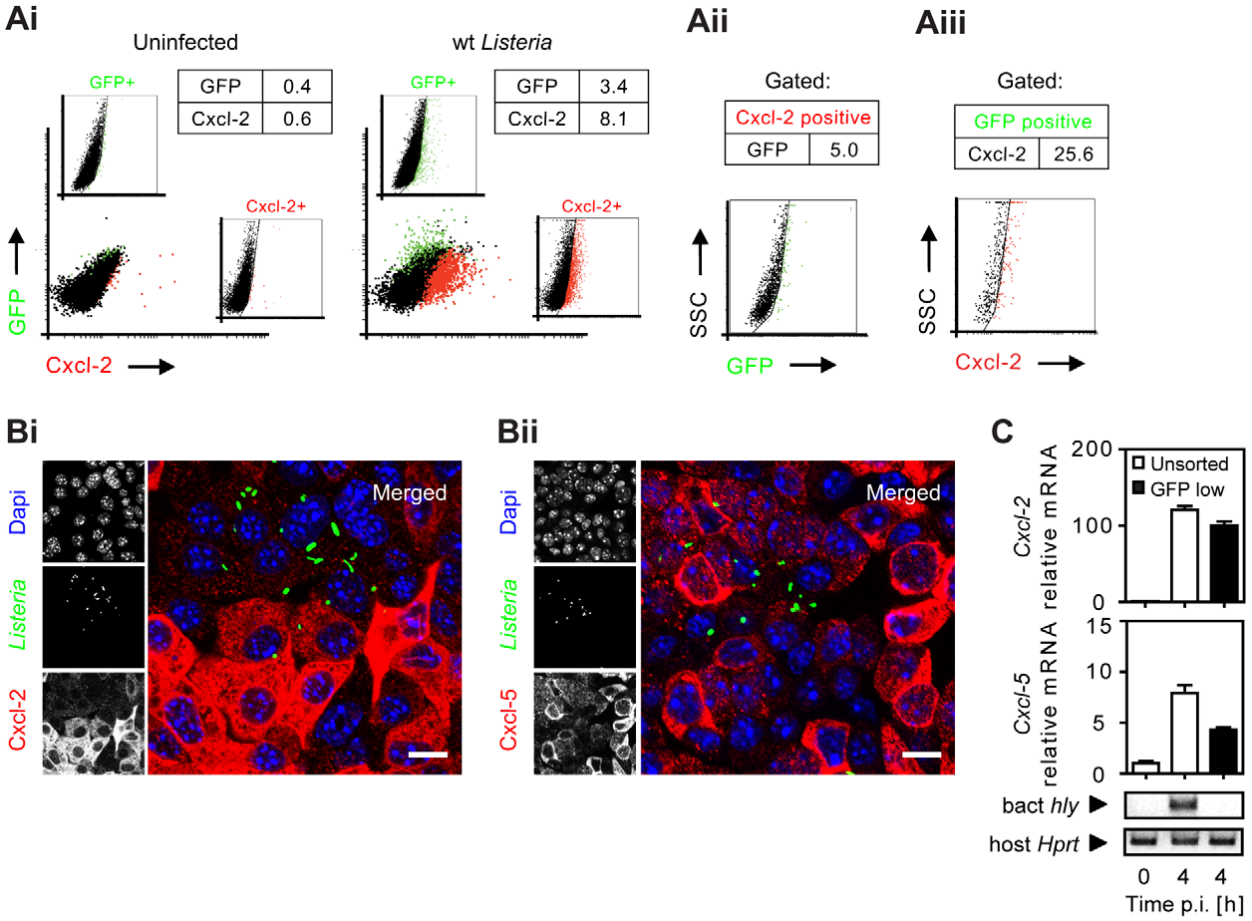
Analysis of bacterial infection and epithelial activation at the single cell level. (**A**) m-IC_cl2_ cells were left uninfected (**Ai**, left) or infected with wt P*_actA_*-*gfp L. monocytogenes* (**Ai**, right). The number [%] of GFP^+^ (*Listeria*-infected, green) or immunolabelled Cxcl-2^+^ (red) cells was visualized 4 h after infection by flow cytometry. Single channel analysis (GFP: FL-1; Cxcl-2: FL-4) was depicted on the side of the axis. (**Aii**) The number of GFP^+^ (*Listeria*-infected) cells among activated, Cxcl-2^+^ cells and (**Aiii**) the proportion of Cxcl-2^+^ cells among GFP^+^ (*Listeria*-infected) cells was demonstrated gating on the respective population. (**B**) m-IC_cl2_ cells were infected with wt P*_actA_*-*gfp L. monocytogenes*. Intracellular *Listeria* (GFP^+^, green) and immunolabelled (**Bi**) Cxcl-2 (red) or (**Bii**) Cxcl-5 (red) was visualized 4 h after infection by fluorescence microscopy. Magnification ×400, counterstaining with Dapi (blue). Scale bar, 5 µm. (**C**) m-IC_cl2_ cells were infected with *actA* mutant *L. monocytogenes* expressing constitutively GFP under control of the *sod* promoter (P*_sod_*-*gfp*). 4 h after infection the GFP**^low^** expressing (*Listeria*-negative) cell fraction was sorted by flow cytometry (see Fig. S2C) and analysed for *Cxcl-2* (upper) or *Cxcl-5* (lower) mRNA expression by RT-PCR. The absence of detectable *Listeria* in the sorted GFP**^low^** cell fraction was demonstrated by PCR amplification of the bacterial *hly* gene (sensitivity limit: 10^3^–10^4^ genome copies). All experiments were performed at a multiplicity of infection of 100∶1. Results are representative for three independent experiments and are presented as mean ± SD (RT-PCR) or show one representative experiment.

To monitor *Listeria* infection and cellular activation simultaneously at the single cell level, bacteria transformed with a vector expressing green fluorescence protein (GFP) either under control of the inducible *actA* promoter [11] or the constitutive *sod* promoter [12] were used for subsequent experiments (for details see Table 1). Surprisingly, flow cytometry revealed that the vast majority of Cxcl-2***^+^*** epithelial cells (95%) were *Listeria*-negative. In addition, only a minor fraction of GFP-positive, *Listeria*-infected epithelial cells exhibited MIP-2 synthesis (Fig. 2A). Similar results were obtained using biotinylated *Listeria* (Fig. S2A). These results were confirmed by immunohistological staining. Cxcl-2 and Cxcl-5 synthesis was not restricted to GFP***^+^*** *Listeria*-infected cells but, in fact, predominantly detected in neighbouring non-infected epithelial cells (Fig. 2B and Fig S2B). Also flow cytometric cell sorting and quantitative RT-PCR analysis strongly supported this unexpected result. A marked upregulation of *Cxcl-2* and *Cxcl-5* mRNA expression was detected in GFP**^low^** expressing (*Listeria*-negative, Fig. S2C) epithelial cells despite the absence of detectable *Listeria* DNA (Fig. 2C). Thus, although epithelial activation requires lysis of the endosomal membrane and contact with cytosolic innate immune receptors, a transcriptional cellular response was mainly observed in non-infected cells.

**Table 1:** *Listeria* strains used in this study.

*L. monocytogenes* EGD	Plasmid	Characteristic	Ref.
wt	-	wild-type strain EGD	[60]
wt	P*_actA_*-*gfp* ^1^	wild-type; *gfp* expression mainly after endosomal lysis	this study
*hly*	-	LLO-deficient: strongly reduced endosomal lysis	[61]
*hly*	P*_actA_*-*gfp* ^1^	LLO-deficient: strongly reduced endosomal lysis; *gfp* expression mainly after endosomal lysis	this study
*hly plcA plcB*		LLO- and PLC-deficient: no endosomal lysis	Chakraborty T.
*actA*		deficient in polar actin accumulation; no lateral cell-to-cell spread	[60]
*actA*	P*_actA_*-*gfp* ^1^	deficient in polar actin accumulation; no lateral cell-to-cell spread, *gfp* expression mainly after endosomal lysis	this study
*actA*	P*_sod_*-*gfp* ^2^	deficient in polar actin accumulation; no lateral cell-to-cell spread,, constitutive GFP expression	this study

P*_actA_* and P*_sod_* are listerial *actA* and *sod* gene promoters, respectively. Abbreviations: *actA*: actin assembly inducing protein precursor; *gfp*: green fluorescent protein; *hly*: listeriolysin O precursor; LLO: listeriolysin O; *plc*: phospholipase C; *sod*: superoxide dismutase.

### Cell-to-cell spread, attachment-induced activation, or listeriolysin are not responsible for indirect cell activation

**Figure 3:**
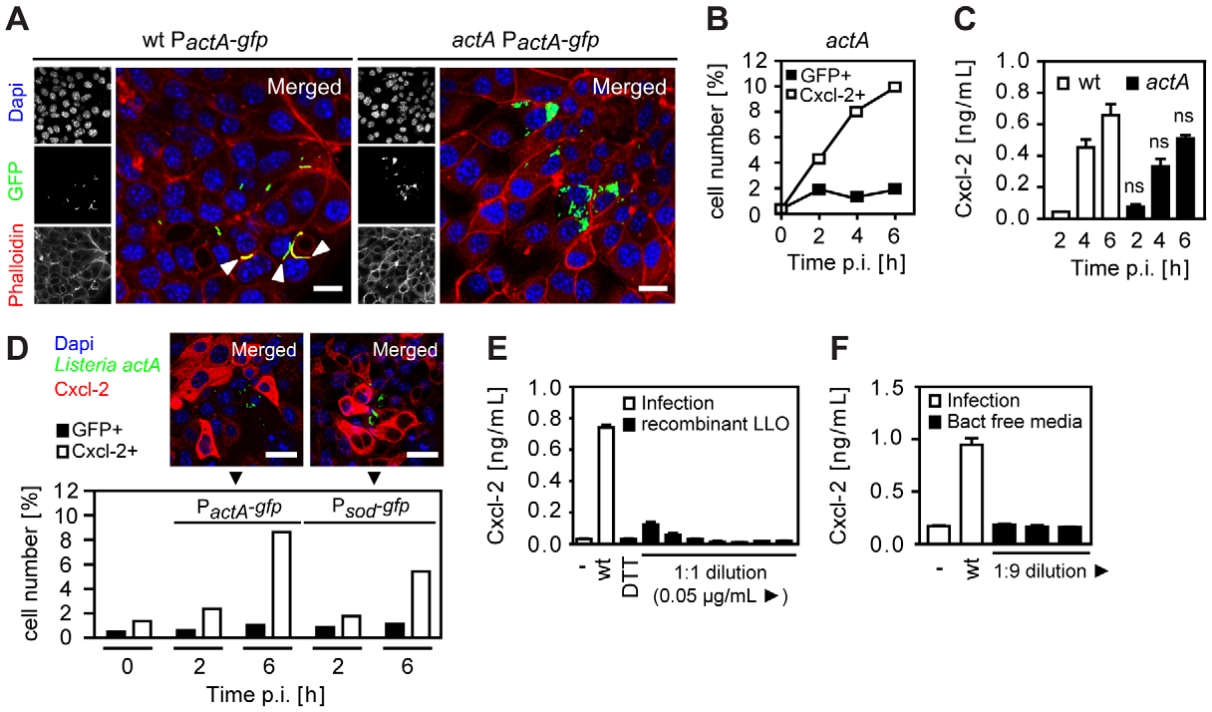
Epithelial activation is not due to bacterial cell-to-cell spread, extracellular attachment, or secreted listeriolysin. (**A**) m-IC_cl2_ cells were infected with wt (left) or *actA* mutant (right) P*_actA_*-*gfp Listeria monocytogenes*. Intracellular *Listeria* (GFP^+^, green) was visualized 6 h after infection by fluorescence microscopy. Magnification ×400, counterstaining with phalloidin (red) and Dapi (blue). White arrows indicate the actin tail assembly by wt *Listeria*. Scale bar, 5 µm. (**B**) m-IC_cl2_ cells were infected with *actA* mutant P*_actA_*-*gfp L. monocytogenes*. The number of GFP^+^ (*Listeria*-infected, black square) or immunolabelled Cxcl-2^+^ (white square) cells was determined after the indicated time by flow cytometry. (**C**) m-IC_cl2_ cells were infected with wt (white) or *actA* mutant (black) *L. monocytogenes*. Cxcl-2 was determined after the indicated time in cell culture supernatant by ELISA; ns, not significant (mutant *versus* wt for the indicated time points). (**D**) m-IC_cl2_ cells were infected with *actA* mutant P*_actA_*-*gfp* or P*_sod_*-*gfp L. monocytogenes*. The number of GFP^+^ (*Listeria*-infected, black) or immunolabelled Cxcl-2^+^ (white) cells was determined after the indicated time by flow cytometry (lower panel). Additionally, intracellular *Listeria* (GFP^+^, green) and immunolabelled Cxcl-2 (red) was visualized 4 h after infection by fluorescence microscopy (upper panel). Magnification ×400, counterstaining with Dapi (blue). Scale bar, 5 µm. (**E**) m-IC_cl2_ cells were infected with wt *L. monocytogenes* or exposed to recombinant listeriolysin at lytic to sublytic concentrations or to the solvent control (DTT). Cxcl-2 was determined 4 h after infection in cell culture supernatant by ELISA. (**F**) m-IC_cl2_ cells were infected with wt *L. monocytogenes* or exposed to undiluted or diluted filtered cultures (bact free media, normalised for multiplicity of infection of 100∶1) of wt *L. monocytogenes* grown in m-IC_cl2_ cell culture media. Cxcl-2 was determined 4 h after infection in cell culture supernatant by ELISA. All infection experiments were performed at a multiplicity of infection of 100∶1. Results are representative for three independent experiments and are presented as mean ± SD (ELISA) or show one representative experiment.

Several mechanisms might account for the observed activation of *Listeria*-negative epithelial cells. Activated epithelial cells might only appear to be *Listeria*-negative due to secondary bacterial escape facilitated by propulsion through ActA-induced actin polymerization in the cytosol and subsequent invasion of the neighbouring cell. Cells primarily infected but secondarily left by lateral spread might thereby appear *Listeria*-negative but in fact would have been previously in contact with cytosolic bacteria (and thus were, in fact, directly activated). To avoid lateral cell-to-cell spread and restrict intraepithelial bacteria to apically infected cells, a *Listeria actA* mutant strain was employed. ActA-deficient *Listeria* exhibited a moderately reduced epithelial invasion (Fig. S3A), a lower percentage of infected epithelial cells, and an enhanced number of bacteria per cell (Fig. 3A and 3B). Nevertheless, high numbers of Cxcl-2 producing epithelial cells (Fig. 3B) and a strong chemokine secretion (Fig. 3C) was observed. Also, the number of activated, Cxcl-2 producing epithelial cells remained significantly higher than the number of *Listeria*-infected cells reaching approximately 10-fold excess of Cxcl-2*^+^* cells (Fig. 3B and Fig. S3B). Thus, indirect activation of epithelial cells was not due to escape from previously infected cells by ActA-driven secondary lateral spread.

Epithelial cell stimulation could also be induced by bacteria either attached to the plasma membrane or remaining intraendosomal and membrane enclosed. To exclude a significant role of attached or intraendosomal *Listeria*, Cxcl-2^+^ and GFP^+^ epithelial cells were quantified after infection with *Listeria* expressing GFP either constitutively under control of the superoxide dismutase promoter (P*_sod_*-*gfp*) or inducible under the control of the *actA* promoter (P*_actA_*-*gfp*) (Table 1). Whereas P*_sod_*-*gfp* carrying *Listeria* exhibited strong reporter expression after growth in bacterial culture medium, only a moderate fluorescence was detected in P*_actA_*-*gfp* –positive bacteria (Fig. S3C). In contrast, strong GFP expression was noted in P*_actA_*-*gfp Listeria* isolated from infected epithelial cells (Fig. S3D). Flow cytometric detection of infected epithelial cells was observed after wt, but not *hly* mutant P*_actA_*-*gfp Listeria* illustrating the endosomal lysis-dependent induction of the *actA* promoter-driven GFP reporter gene expression (Fig. S3E). Infection with P*_sod_*-*gfp* or P*_actA_*-*gfp* carrying wt *Listeria* resulted in a significant number of *Listeria*-infected epithelial cells. Importantly, a higher number of Cxcl-2*^+^* activated cells as compared to *Listeria*-infected cells was observed by flow cytometry after infection with both reporter constructs and epithelial Cxcl-2 synthesis was similarly noted in *Listeria*-negative epithelial cells (Fig. 3D). These results suggest that indirect epithelial activation was not a result of attached or intraendosomal bacteria [13].

Finally, the activation of *Listeria*-negative epithelial cells might be due to the stimulatory effect of secreted bacterial molecules, such as the cytolytic listeriolysin O (LLO) [14], [15]. Therefore, the membrane damaging as well as the stimulatory effect of recombinant listeriolysin (rLLO) on red blood cells (RBC) and epithelial m-IC_cl2_ cells was analysed. High concentrations of rLLO induced significant hemoglobin and detectable lactate dehydrogenase (LDH) release by RBCs and epithelial m-IC_cl2_ cells, respectively (Fig. S3F and Fig. S3G). Quantitation of epithelial cell activation in response to rLLO, however, revealed an only minor response as compared to epithelial Cxcl-2 secretion after viable wt *L. monocytogenes* infection (Fig. 3E). Similarly, no significant Cxcl-2 secretion by epithelial cells was noted in response to bacteria-free culture supernatant derived from *Listeria* cultures with bacterial counts precisely corresponding to the infection model described above (Fig. 3F). Yet, culture supernatants derived from wild-type or ActA-deficient bacteria exhibited significant hemolytic activity, in contrast to supernatant from *hly*-deficient *Listeria* (Fig. S3H). No significant membrane damage was noted after infection of intestinal epithelial cells with wt, *actA*, or *hly* mutant *Listeria* (Fig. S3I). Although a supportive effect of released bacterial factors cannot be excluded, these results suggest that bacterial mediators do not play a major role in the observed indirect epithelial activation. Thus, neither basolateral cell-to-cell spread nor membrane attachment, or the secretion of LLO in the cell culture supernatant appear to be responsible for indirect epithelial cell activation after *L. monocytogenes* infection. This suggests the presence of a previously unrecognized mechanism of epithelial intercellular communication in response to bacterial infection.

### Indirect epithelial activation is not induced by epithelial transcriptional activation *per se*

To examine whether indirect epithelial stimulation by horizontal cell-to-cell communication might be a general effect of transcriptional activation of intestinal epithelial cells, a bicistronic expression vector encoding the NF-κB subunit RelA/p65 together with GFP under the control of a constitutive cytomegalovirus (CMV) promoter was employed (Fig. S4A). Transient overexpression of RelA/p65 alone or bicistronic expression of RelA/p65 and GFP readily induced epithelial activation as illustrated by NF-κB reporter gene upregulation (Fig. S4B) and enhanced chemokine secretion (Fig. S4C). Although RelA/p65-mediated Cxcl-2 production exhibited a slower kinetic as compared to following *Listeria* infection, a significant number of Cxcl-2^+^ cells was detected. Of note, RelA/p65-mediated cellular activation was restricted to GFP^+^, i.e. directly activated epithelial cells (Fig. S4D). Cxcl-2 production by GFP^+^ cells increased strongly (0.1 *versus* 2.4%), whereas the number of Cxcl-2^+^ cells in the GFP^-^ population remained virtually unchanged (0.8% *versus* 1.2%). In addition, the number of Cxcl-2^+^ epithelial cells did not exceed the number of transfected GFP^+^ cells at any time (Fig. S4E). Thus, epithelial activation *per se* does not induce indirect cell activation by horizontal intercellular communication. Indirect epithelial activation appears rather to be induced by innate immune signal transduction upstream of transcription factor activation.

### Analysis of cytokine secretion, ion channel stimulation, and gap junction activity in horizontal cell-cell communication

**Figure 4:**
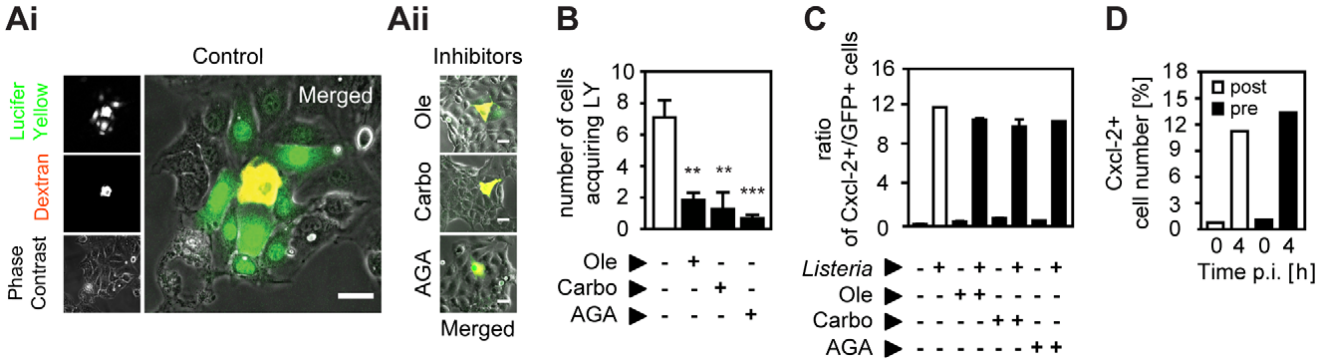
Inhibition of gap junctional transport, cytokine and prostaglandin secretion, or ion fluxes does not affect epithelial intercellular communication. (**A**) m-IC_cl2_ cells were microinjected with a mixture of Texas Red-conjugated dextran (red) and the gap junction permissible Lucifer Yellow in the absence (**Ai**, Control) or presence (**Aii**) of the gap junction inhibitor oleamide (Ole, 0.1 mM), carbonoxolone (Carbo, 0.01 mM), or α-glycerrhetinic acid (AGA, 0.1 mM). The cellular spread of Lucifer Yellow was visualized 5 min after microinjection by live imaging fluorescence microscopy. Magnification ×400. Phase contrast images were added to visualize single cells. Scale bar, 5 µm. (**B**) Number of cells acquiring Lucifer Yellow (LY) by gap junction mediated transport after microinjection in the absence or presence of gap junction inhibitors. A minimum of 10 microinjected cells were analysed per experiment; **, p<0.01, ***, p<0.005. (**C**) m-IC_cl2_ cells were infected with *actA* mutant P*_actA_*-*gfp L. monocytogenes*. The ratio of immunolabelled Cxcl-2^+^ cells to GFP^+^ (*Listeria*-infected) cells was determined 4 h after infection in the absence or presence of gap junction inhibitors by flow cytometry. (**D**) m-IC_cl2_ cells were infected with *actA* mutant P*_sod_*-*gfp L. monocytogenes*. The number of immunolabelled Cxcl-2***^+^*** cells was determined after the indicated time by flow cytometry. Brefeldin A was added 60 min after (post, white bars) or 30 min prior to (pre, black bars) infection. All infection experiments were performed at a multiplicity of infection of 100∶1. Results are representative for three independent experiments and are presented as mean ± SD (microinjection) or show one representative experiment.

Next we investigated the mechanism underlying horizontal cell-to-cell communication and coordinated epithelial chemokine upregulation in response to *Listeria* infection. Functional gap junctional transport was examined by microinjection of transferable Lucifer Yellow together with non-transferable high molecular weight dextran. Fluorescence imaging visualized transport of Lucifer Yellow from the microinjected cell to the surrounding neighbouring cells. Addition of inhibitors of gap junctional transport, effectively reduced lateral diffusion of Lucifer Yellow after microinjection (Fig. 4A and B). Inhibition of gap junctional intercellular communication, however, did not decrease the number of activated epithelial cells after *Listeria* infection as illustrated by the unaltered high ratio of activated (Cxcl-2^+^) to infected (GFP^+^) epithelial cells measured by flow cytometry (Fig. 4C). Although these results do not completely rule out transfer of very small signaling molecules by gap junctional transport channels, they do not support a major role in the process of horizontal communication.

Similarly, the potential role of a secreted protein messenger was examined. Intestinal epithelial m-IC_cl2_ cells were exposed to brefeldin A (BFA), an effective inhibitor of the secretion of newly synthesized proteins (Fig. S4F), prior and after infection with *actA* mutant *L. monocytogenes*. The number of *Listeria*-induced Cxcl-2^+^ cells, however, was not altered irrespective whether BFA was administered 30 min prior or 60 min after infection (Fig. 4D). Second, cell culture medium was obtained 10, 20, 30, 40, or 60 min after *Listeria* infection, centrifuged to remove bacteria, and immediately transferred to naïve uninfected epithelial cells. Yet no epithelial activation was observed after exposure to conditioned culture supernatant despite significant Cxcl-2 synthesis detected in the *Listeria* infected cell population (Fig. S4G). Of note, factors released by *Listeria*-infected cells might be unstable or immediately bound to neighbouring cells preventing their efficient release in the conditioned cell culture supernatant.

Finally, widely used pharmacological inhibitors of prostaglandin synthesis and known intestinal epithelial ion channels were employed. Indomethacin, an inhibitor of cyclooxygenase isoenzymes (COX1, COX2) involved in prostaglandin synthesis, thapsigargin, an inhibitor of the endoplasmatic Ca^2+^ATPase, CFTR II, a selective apical Cl^−^ ion channel inhibitor, and bumetanide, an inhibitor of a basolateral epithelial Na^+^K^+^Cl^−^ cotransporter had no significant influence on the number of activated epithelial cells after *Listeria* infection illustrated as ratio of activated (Cxcl-2^+^) to infected (GFP^+^) cells (Fig. S4H). These results do not identify a significant role of gap junctional transport, secreted protein or prostaglandin mediators, or ion fluxes in the observed indirect activation of epithelial cells after *L. monocytogenes* infection.

### *Listeria*-induced horizontal epithelial communication depends on oxygen radical synthesis

**Figure 5:**
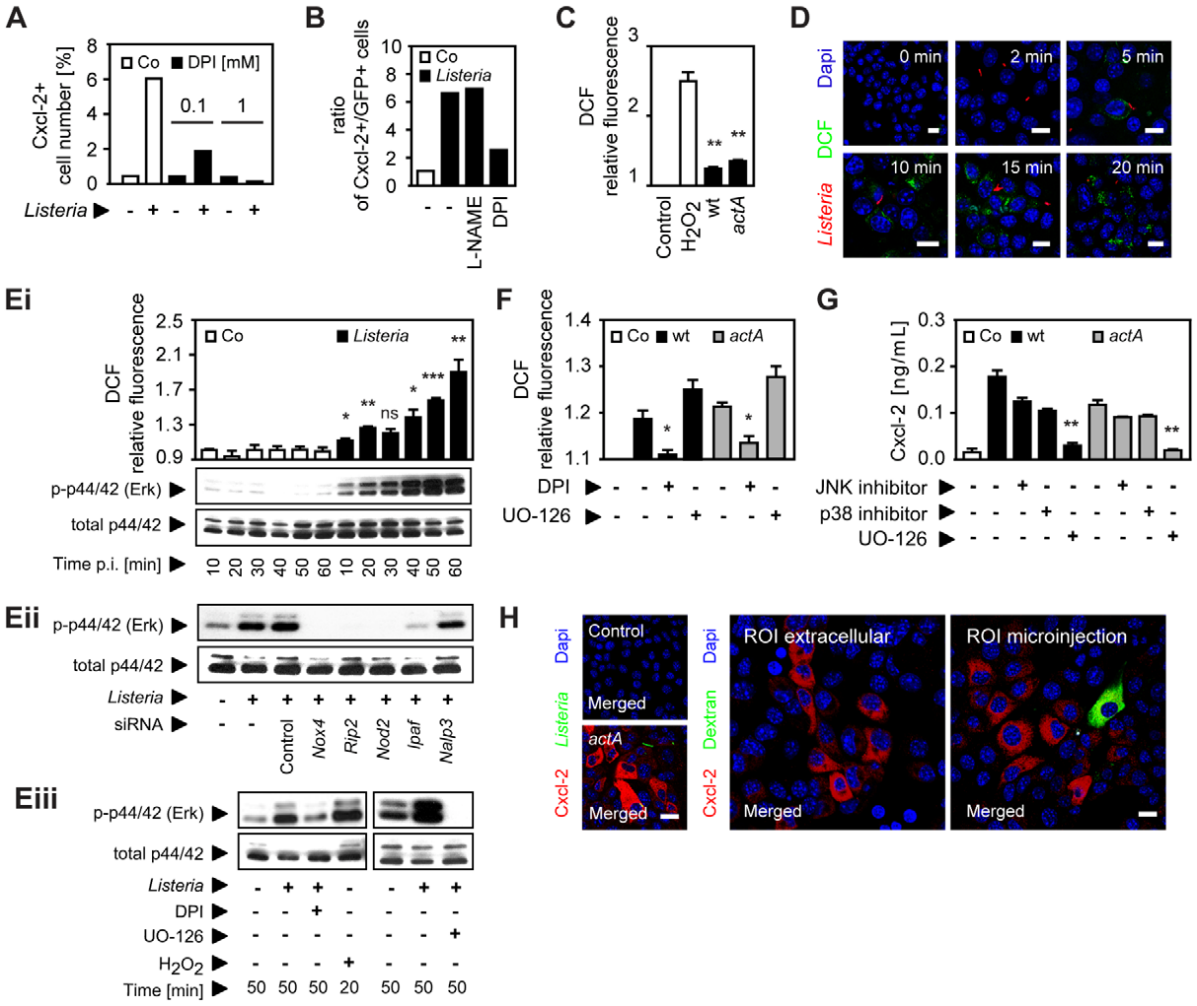
Epithelial intercellular communication is dependent on *Listeria*-induced oxygen radical synthesis. (**A**) m-IC_cl2_ cells were infected with *actA* mutant P*_actA_*-*gfp Listeria monocytogenes*. The number of immunolabelled Cxcl-2^+^ cells was determined 4 h after infection in the absence or presence of diphenylene iodonium (DPI, mM) by flow cytometry. (**B**) m-IC_cl2_ cells were infected with *actA* mutant P*_actA_*-*gfp L. monocytogenes*. The ratio of immunolabelled Cxcl-2^+^ cells to GFP^+^ (*Listeria*-infected) cells was determined 4 h after infection in the absence or presence of N (G)-nitro-L- arginine methyl ester (L-NAME) or DPI (each at 0.1 mM) by flow cytometry. (**C**) m-IC_cl2_ cells were loaded with DCF-DA and infected with wt or *actA* mutant *L. monocytogenes*. DCF relative fluorescence, reflecting reactive oxygen intermediates (ROI) production was quantified 20 min after infection by fluorescence spectroscopy. H_2_O_2_ (1 mM) was used as positive control. (**D**) m-IC_cl2_ cells were loaded with DCF-DA and infected with immunolabelled wt *L. monocytogenes* (red). DCF fluorescence, reflecting ROI production was visualized at the indicated time after infection by fluorescence microscopy. Magnification ×400, counterstaining with Dapi (blue). Scale bar, 5 µm. (**Ei**) m-IC_cl2_ cells were loaded with DCF-DA and infected with wt *L. monocytogenes*. DCF relative fluorescence, reflecting ROI production (upper panel) or p44/42 (Erk) phosphorylation (lower panel) was determined at the indicated time after infection by fluorescence spectroscopy or by immunoblotting, respectively. (**Eii**) m-IC_cl2_ cells were treated with small interfering RNA (siRNA) control or siRNA *Nox4*, *Rip2*, *Nod2*, *Ipaf* or *Nalp3* and infected with wt *L. monocytogenes*. p44/42 phosphorylation was determined 50 min after infection by immunoblotting. (**Eiii**) m-IC_cl2_ cells were infected with wt *L. monocytogenes* in the absence or presence of DPI (0.1 mM) or exposed to H_2_O_2_ (1 mM) [left], or alternatively in the absence or presence of UO-126 (10 µM) [right]. p44/42 phosphorylation was determined after the indicated time by immunoblotting. (**F**) m-IC_cl2_ cells were loaded with DCF-DA and infected with wt (black square) or *actA* mutant (gray square) *L. monocytogenes*. DCF relative fluorescence, reflecting ROI production was quantified 20 min after infection in the absence or presence of DPI (0.1 mM) or UO-126 (10 µM) by fluorescence spectroscopy. (**G**) m-IC_cl2_ cells were infected with wt (black square) or *actA* mutant (gray square) *L. monocytogenes*. Cxcl-2 was determined 4 h after infection in the absence or presence of the indicated inhibitors in cell culture supernatant by ELISA. Used inhibitors: JNK inhibitor (1 µg/mL), p38 inhibitor (2 µg/mL), UO-126 (10 µM). (**H**) m-IC_cl2_ cells were left untreated (left, upper), infected with *actA* mutant P*_actA_*-*gfp L. monocytogenes* (GFP^+^, green, left, lower), or exposed to cumene hydroperoxide (0.5 mM) in cell culture medium (ROI extracellular, middle panel), or microinjected with cumene hydroperoxide (ROI microinjection, 0.5 mM, left panel). Immunolabelled Cxcl-2 (red) was visualized 4 h after infection or stimulation by fluorescence microscopy. The microinjected cell was loaded with Texas Red-conjugated dextran (green). Magnification ×400, counterstaining with Dapi (blue). Scale bar, 5 µm. All experiments were performed at a multiplicity of infection of 100∶1. Results are representative for three independent experiments and are presented as mean ± SD (ROI, ELISA) or show one representative experiment. *, p<0.05, **, p<0.01, ***, p<0.005, ns, not significant.

Since unstable and highly reactive host-derived factors were not excluded by the previous experiments, a possible involvement of oxygen or nitrogen radicals in horizontal epithelial cell-cell communication was subsequently evaluated. Expression of members of two enzyme families, NADPH oxidases and nitric oxide synthase (NOS), has been described in epithelial cells [16]. Indeed, addition of the NADPH oxidase inhibitor diphenylene iodonium (DPI) resulted in a significant reduction of *Listeria*-induced epithelial activation (Fig. 5A). DPI did not reduce *Listeria* survival in epithelial cells (Fig. S5B) and had no effect on LPS or PMA-induced epithelial activation (Fig. S5A). In contrast to DPI, the NOS inhibitor N (G)-nitro-L- arginine methyl ester (L-NAME) did not influence the number of activated epithelial cells (Fig. 5B). In accordance with an inhibitory effect of DPI, synthesis of reactive oxygen intermediates (ROI) after *Listeria* infection was observed (Fig. 5C). ROI was detected in focal areas of confluent epithelial cells surrounding *Listeria*-positive, infected cells in accordance with local production and lateral spread of ROI as early as 10 min after infection (Fig. 5D).

Innate immune receptor stimulation by *Listeria* infection of epithelial cells resulted in rapid activation of the mitogen-activated protein (MAP) kinase Erk in a ROI-dependent manner (Fig. 5E). Whereas impairment of the MAP kinase Erk had no significant effect on ROI production (Fig. 5F), *Listeria*-induced Cxcl-2 synthesis by intestinal epithelial cells was completely abrogated by Erk inhibition and partially also dependent on the MAP kinases p38 and JNK (Fig. 5G). Of note, Erk inhibition did not affect bacterial invasion and the viability of intracellular *Listeria* (Fig. S5B). Finally, exposure of epithelial cells to cumene hydroperoxide, a ROI liberating organic agent within the cell culture medium or by microinjection induced Cxcl-2 synthesis in neighbouring cells similar to *L. monocytogenes* infection (Fig. 5H). Thus, *Listeria*-infection induces significant epithelial ROI synthesis, which in turn mediates MAP kinase Erk activation and downstream Cxcl-2 production.

**Figure 6:**
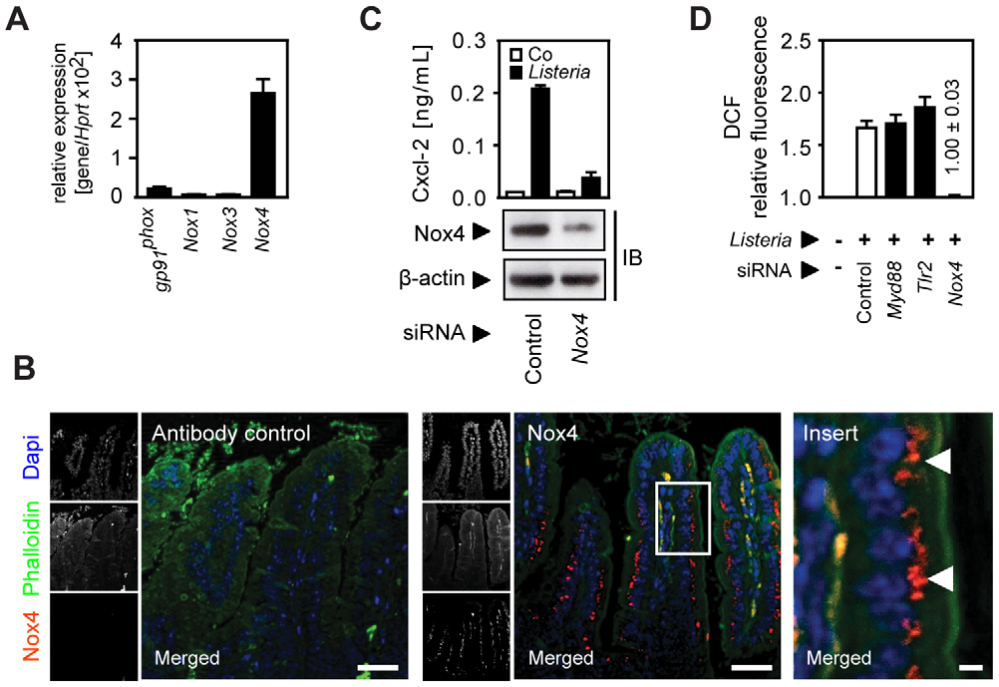
Activation of the NADPH oxidase (Nox) 4 mediates indirect epithelial activation. (**A**) Expression of *gp91* ***^phox^*** (*Nox2*), *Nox1*, *3*, and *4* in isolated highly pure (>98% E-cadherin^+^, CD45^−^) primary intestinal epithelial cells. (**B**) Immunostaining for Nox4 expression in sections of C57BL/6 mouse small intestinal tissue. Magnification ×200; counterstaining with phalloidin (green) and Dapi (blue). Scale bar, 50 µm. For demonstration of the subcellular localisation of Nox4 (red), the insert (white frame) was enlarged ×630 (right panel). White arrows highlight the subcellular localization of Nox4. Scale bar, 5 µm. (**C**) m-IC_cl2_ cells were treated with small interfering RNA (siRNA) control or siRNA *Nox4* and left uninfected (white) or were infected (black) with *actA* mutant *L. monocytogenes*. Cxcl-2 was determined 4 h after infection in cell culture supernatant by ELISA (upper panel). Nox4 downregulation was verified by immunoblotting (lower panel). (**D**) m-IC_cl2_ cells were treated with small interfering RNA (siRNA) control or siRNA *MyD88*, *Tlr2*, or *Nox4*, loaded with DCF-DA and infected with *actA* mutant *L. monocytogenes*. DCF relative fluorescence, reflecting ROI production, was quantified 20 min after infection by fluorescence spectroscopy. All infection experiments were performed at a multiplicity of infection of 100∶1. Results are representative for three independent experiments and are presented as mean ± SD (ELISA, ROI) or show one representative experiment.

Oxygen radical synthesis is performed by an oligomeric protein complex involving a cell type-specific NADPH-oxidase (Nox) protein. Only significant expression of the Nox4 isoform was detected in primary small intestinal epithelial cells (Fig. 6A). Nox4 synthesis was restricted to intestinal epithelial cells as demonstrated by immunostaining with a paranuclear expression pattern in accordance with a previous report (Fig. 6B) [16]. Importantly, downregulation of *Nox4* expression in epithelial cells by siRNA interference significantly reduced Cxcl-2 secretion (Fig. 6C) and ROI production upon *Listeria*-infection (Fig. 6D). In contrast, downregulation of *Nox4* expression did not alter LPS- or PMA-induced chemokine secretion (Fig. S6). Thus, ROI production by Nox4 appears to be both necessary and sufficient to induce horizontal cell-cell communication in intestinal epithelial cells leading to chemokine secretion in neighbouring cells in response to *Listeria* infection.

## Discussion

Communication between individual cells is a fundamental feature of multicellular organisms. For instance, it mediates a coordinated reaction of muscle cell contraction, and allows neuronal signal transmission or endocrinological regulatory circuits. Cell-cell communication is also characteristic for the complex regulatory networks of the adaptive immune system. Cytokines bridge anatomical distances to coordinate and amplify the host response against pathogens. In the present study we investigated whether cell-cell communication between neighbouring cells might also contribute to innate immune activation within a confluent epithelial cell layer to coordinate the antimicrobial host defence at an early stage of the infection. Although inhibition of the overall epithelial responses by interference with the production of soluble mediators and gap junction integrity had previously been noted, the process of immune stimulation and cellular response upon bacterial infection has not been studied at the single cell level [17], [18], [19], [20], [21]. The present study therefore represents the first report to demonstrate epithelial horizontal cell-cell communication upon bacterial innate immune stimulation.

For three reasons, *Listeria* infection of confluent intestinal epithelial cells represents an ideal model to study epithelial cell-cell communication downstream of innate immune stimulation. First, similar to other pathogenic bacteria *Listeria monocytogenes* escapes from the endosomal vacuole and proliferates within the host cell cytosol [6]. Endosomal escape is associated with a dramatic change in bacterial gene expression. Although expressed at low levels also during in vitro culture, a very strong upregulation of the actin polymerizing protein ActA provides an excellent reporter for detection of cytosolic entry [22]. Second, bacteria lacking *hly*, *plcA*, or *plcB* mediating endosomal lysis only induce an only minor activation which might result from intraendosomal recognition or a so far unidentified minor mechanism of endosomal escape. Thus, in contrast to macrophages that recognize *Listeria* also at the plasma membrane, epithelial cell stimulation is mainly observed when bacteria reach the cytosol, facilitating contact with cytosolic innate immune receptors such as Ipaf, Nalp3, and Nod2 [9], [10]. This finding excludes innate immune recognition and receptor-mediated initiation of signal transduction in non-infected, *Listeria*-negative cells. Third, one amino acid exchange between the mouse and human E-cadherin causes a strongly reduced infection rate in murine epithelial cells [23], leaving most cells of a confluent cell layer uninfected and accessible to the analysis of indirect cellular activation. Using reporter gene technology, intracellular chemokine staining and flow cytometric analysis, we were able to demonstrate that the chemokine secretion in response to *Listeria* infection is mainly derived from uninfected, indirectly activated epithelial cells. Of note, the commonly used quantification of cytokine secretion in the cell culture supernatant or immunoblotting of total cell lysate proteins would not have disclosed this surprising finding.

Epithelial stimulation on the transcriptional level by p65/RelA overexpression did not result in detectable indirect cell activation. Several possible mechanisms of horizontal cell-cell communication downstream of innate immune receptor signaling were therefore considered. In response to microbial stimulation, epithelial cells produce chemokines, prostaglandins, and cause local alterations of ion concentrations by regulating transmembrane ion channel activity. Also, gap junctional intercellular communication (GJIC) represents a direct cytosolic connection and might be used to forward the information of innate immune recognition within the epithelial cell layer [17], [21]. Ca^2+^ fluxes via intercellular gap junctions have been shown to promote lung epithelial chemokine secretion [18] and intact gap junction formation has also been linked to innate immune stimulation and maintenance of the epithelial barrier [19]. On the other hand, connexin-26 hemichannel-mediated Ca^2+^ signaling has also been proposed to promote bacterial invasion and lateral spread [24]. Yet, neither protein secretion, nor ion channel activity or gap junction formation appeared to be involved in *Listeria*-induced indirect epithelial cell activation.

Instead, our results indicate an important role of reactive oxygen intermediates (ROI) in horizontal epithelial cell-cell communication. ROI represent reduction products of molecular oxygen such as the radical superoxide (•O_2_^−^) and hydroxyl (•OH), and the non-radical hydrogen-peroxide (H_2_O_2_). ROI production by professional phagocytes during oxidative burst provides significant bactericidal activity but synthesis is also observed in non-phagocytic cells [25]. ROI at subtoxic doses has been recognized as an important intracellular signal transducing molecule during the recent years [26], [27], [28], [29]. In accordance with our results ROI-induced activation of MAP kinase activity has been reported [30], [31], [32], [33]. In addition, an involvement of ROI in the cellular signaling leading to NF-κB activation [34], apoptosis [31], [35], epidermal growth factor receptor signaling [36], regulation of cellular proliferation [37], and antimicrobial peptide production [38], [39] has been described. ROI was also shown to prime *Drosophila melanogaster* hematogenic progenitor cells for differentiation [40] and to play an important role in the fruit fly's intestinal immunity [41]. Whereas the half-life of oxygen radical hydroxyl (•OH) is extremely short (10^−9^s) and the superoxide •O_2_^−^ is membrane impermeable [40], H_2_O_2_ is able to diffuse to neighbouring cells and induce cellular activation. Indeed, a tissue gradient of H_2_O_2_ was shown to induce rapid recruitment of leukocytes into the wound margin following endothelial hypoxia [42], [43].

NADPH oxidase activation has previously been linked to innate immune mediated antimicrobial killing [25] as well as receptor signal transduction [44], [45], [46], [47], [48], [49], [50], [51]. Here we for the first time report Nox4 expression by intestinal epithelial cells and demonstrate Nox4-mediated ROI production in response to bacterial infection. Although enhanced *Nox4* mRNA expression was shown to result in increased ROI production [52], the initiation of Nox4-dependent ROI production upon *Listeria* infection was noted as early as 5–10 minutes after bacterial challenge. This excludes a significant role of transcriptional regulation of Nox4 in our model. Whereas the prototypical NADPH oxidase of phagocytes, gp91*^phox^* (Nox2), requires cytosolic proteins such as p47*^phox^* to form a functional NADPH oxidase complex, Nox4 functions independent of cytosolic accessory proteins. Interestingly, Nox expression has previously been linked to innate immune receptor signaling: Nox4 activation was shown to be involved in TLR4-mediated NF-κB activation in human epithelial kidney cells and monocytes [47], [53]. In contrast, our results revealed activation of intestinal epithelial cells by *Listeria* infection in a Nod2-, Ipaf-, and Nalp3-dependent fashion which was followed by ROI production and subsequent MAP kinase signaling. Our data are therefore in accordance with previous reports on MAP kinase activation after Nox4-mediated ROI production [54], [55]. A future analysis of the local paracellular concentration of the different species of oxygen radicals might help to improve our understanding of the regulatory role of Nox4-mediated ROI production for epithelial cell-cell communication. Interestingly, reduced chemokine synthesis was noted in directly infected, *Listeria*-positive cells. These cells were also impaired to respond to secondary innate immune stimulation illustrating the immune evasive behaviour of *L. monocytogenes* (data not shown). Although the underlying mechanism is currently not resolved, high concentrations of ROI were previously associated with reduced susceptibility to immunostimulatory agents [56]. Yet other bacterial or host factors such as antioxidant enzymes might reduce local ROI concentrations and interfere with cellular activation and chemokine production in infected epithelial cells.

**Figure 7:**
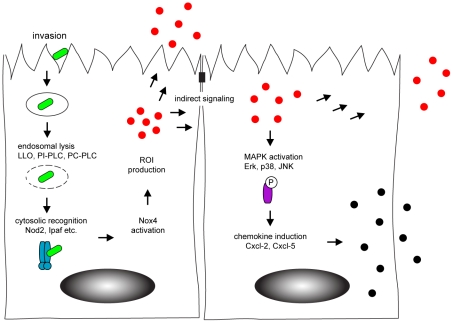
Model of horizontal intercellular communication between intestinal epithelial cells. Following bacteria-induced internalization, *Listeria monocytogenes* (green) escapes from the endosome by listeriolysin (LLO) and phospholipase-mediated rupture. This facilitates recognition by cytosolic innate immune receptors (blue) that activate the NADPH oxidase (Nox) 4 and induce the production of the reactive oxygen intermediates (red). Reactive oxygen intermediates contribute to the activation of uninfected, neighbouring cells. Indirect stimulation subsequently initiates the MAPK-dependent secretion of chemokines (black) to attract professional immune cells to the site of infection.

In conclusion, our data for the first time analyzed intestinal epithelial activation in response to bacterial infection at a single cell level. We could detect Nox4 expression by intestinal epithelial cells which facilitated rapid ROI production upon infection and paracrine activation of neighbouring cells (Fig. 7). Our findings thus identify horizontal cell-cell communication to allow a coordinated innate immune activation of the intestinal epithelium. The present work significantly broadens our knowledge on the complex processes that underlie mucosal innate immune stimulation and illustrates the specific role of epithelial cells for an efficient activation of the antimicrobial host defence.

## Materials and Methods

### Antibodies and reagents

Intracellular Cxcl-2 (MIP-2) and Cxcl-5 was detected using rabbit antibodies from Nordic Biosite (Täby, Sweden). The rabbit polyclonal anti-actin antiserum was from Sigma-Aldrich (Taufenkirchen, Germany). The rabbit-anti-mouse Nox4 antiserum was obtained by immunization with recombinant peptide. The rabbit anti-p-p44/42 (phospho-Erk) and the mouse anti-p44/42 (total-Erk) was from Cell Signaling Technology (Beverly, MA, USA). The rabbit anti-*Listeria* antibody was from Dunn Labortechnik GmbH (Asbach, Germany). Cy5-, Cy3-, HRPO-conjugated secondary antibodies were from Jackson ImmunoResearch (West Grove, PA, USA) and the Alexa Fluor (AF) 488-, and AF 555-conjugated donkey anti-rabbit IgG (H+L) was from Invitrogen (Molecular Probes). The MFP590- and MFP488-labelled phalloidin were purchased from MoBiTec GmbH (Goettingen, Germany). The Sulfo NHS-LC-Biotin was obtained from Pierce, Thermo Scientific (Rockford, IL, USA). *Escherichia coli* K12 D31m4 LPS was ordered from List Biological Laboratories (Campbell, CA, USA). Recombinant LLO (rLLO) was expressed and purified exactly as described before [57]. rLLO was applied to cells in a serial dilution with 0.05 µg/mL as highest concentration. Cxcl-2 was quantified using an ELISA from Nordic Biosite or R&D Systems (Quantikine, R&D Systems GmbH, Wiesbaden, Germany). The NF-κB reporter construct pBIIX-luciferase carrying two copies (2× NF-κB) of the κB sequences from the Igκ enhancer was provided by S. Ghosh (Yale University Medical School, New Haven, CT, USA). Luciferase activity was quantified with luciferin substrate (PJK GmbH, Kleinblittersdorf, Germany). The bicistronic RelA/p65 expression plasmid was cloned by removing the nef gene from a pCG-nef-IRES-GFP expression plasmid (provided by J. Muench, Institute of Virology, University Clinic of Ulm, Germany) by digestion with the restriction enzymes XbaI and MluI (Fermentas, St Leon-Rot, Germany) and replacing it *in frame* with the p65 encoding gene amplified from a p65 expression plasmid (obtained from by U. Pahl, University Clinic, Freiburg, Germany) using the forward: 5′-ACC TCT AGA CCA TGG ACG ATC TGT TTC C-3′ and reverse: 5′-ACG ACG CGT GCA CCT TAG GAG CTG ATC TGA-3′ primers and digested with XbaI/MluI prior to ligation. Plasmid DNA for transfection was prepared using the endotoxin-free plasmid kit from Qiagen (Hilden, Germany). Targeted siRNA probes (*Tlr2*, *Rip2*, *Nox4*, *Card1*2, *Cias1*, control siRNA,) were from Qiagen (Hilden, Germany), the *Card15* siRNA was from Santa Cruz (Heidelberg, Germany). Lipofectamin 2000 (Invitrogen, Carlsbad, CA, USA) and INTERFERin (Polyplus Transfection, New York, NY, USA) were used for plasmid and siRNA transfection, respectively. The pharmacological inhibitors and radical donors oleamide, carbonoxolone, α-glycerrhetinic acid, brefeldin A, thapsigargin, CFTR inhibitor II (CFTR II.), indomethacin, bumetanide, N-(G)-nitro-L- arginine methyl ester (L-NAME), UO-126, hydrogen peroxide (H_2_O_2_) and cumene hydroperoxide were purchased from Sigma Aldrich. The p38 inhibitor 3-O-Acetyl-beta-boswellic acid and the L-stereoisomer JNK inhibitor 1 were from Enzo Life Sciences (Lörrach, Germany), and diphenylene iodonium (DPI) from Cayman Chemical (Hamburg, Germany). Defibrinated sheep red blood cells (SRBC) were purchased from Oxoid (Basingstoke, UK). The LDH Cytotoxicity Assay Kit was from Cayman Chemical (Hamburg, Germany). Colorimetric (ELISA, LDH), luminescent (luciferase) and fluorescent (ROI) measurements were carried out using a Victor3 Multilabel Plate Reader (Perkin Elmer, Waltham, MA, USA). Cell culture reagents were purchased from Invitrogen. All other reagents were obtained from Sigma Aldrich (Taufkirchen, Germany) if not stated otherwise.

### Cell culture and stimulation assays

The m-IC_cl2_ small intestinal epithelial cell line has previously been described [58]. Cells were cultured in a modified, hormonally defined medium with DMEM and F12 (vol 1∶1) supplemented with 5% FCS, 2% glucose, 20 mM Hepes, 2 mM glutamin, 5 µg/mL insulin, 50 nM dexamethasone, 60 nM sodium selenite, 10 ng/mL epithelial growth factor, 5 µg/mL transferrin, and 1 nM 3,3′,5-triiodo-L-thyronine sodium salt. Cell passages 42–70 were used. Cells were grown at 37°C in a 5% CO_2_ atmosphere on collagen-coated cell culture plates or chambers to reach a polarized, confluent monolayer. Rat tail collagen was ordered from Institut Jacques Boy (Reims, France). Specific targeted or control siRNA was transfected at a final concentration of 10 nM 36 hours prior to functional analysis. Stimulation with lipopolysaccharide (LPS) was performed at a final concentration of 10 ng/mL.

### Bacterial strains and epithelial infection

*Listeria monocytogenes* EGD wild-type (wt), *actA*, *hly* deletion mutant strains and the *hly*/*plcA*/*plcB* triple mutant strain are described in Table 1. Fluorescent bacteria were generated by transformation [59] with GFP expression vectors under the control of the *actA* or *sod* promoter (P*_actA_*-*gfp*, P*_sod_*-*gfp*; Table 1) Bacteria were routinely grown in Brain Heart Infusion (BHI) broth, supplemented with antibiotics when required. Overnight cultures were diluted 1∶50, grown to middle logaritmic phase (OD_600_) with mild agitation at 37°C, washed, and added in cell culture medium at the multiplicity of infection (m.o.i.) of 100∶1 (if not stated otherwise) followed by centrifugation (1500 rpm, 5 min, 4°C). 60 minutes after addition of bacteria, epithelial monolayers were washed three times with PBS, and fresh medium containing 50 µg/mL gentamycin was added to the culture medium to restrict extracellular bacterial growth. Unless indicated otherwise, infections were completed after 4 h post infection. To quantify bacterial invasion, co-culture of 20, 40 or 60 min was followed by 1 h incubation in fresh cell culture medium supplemented with 50 µg/mL gentamycin. For 4 h and 6 h infection, gentamycin was supplemented 60 minutes after addition of bacteria, and incubation was carried out for additional 3 h or 5 h. After washing, cells were lysed in 0.1% Triton/H_2_O and the number of intracellular bacteria was determined (CFU) by serial dilution and plating. Bacteria free conditioned medium were prepared by centrifugation or filtering of cell culture medium, and immediately applied on naïve, uninfected m-IC_cl2_ cells. The rabbit anti-*Listeria* antibody was used for immunolabelling of bacteria (1∶500). For alternative intracellular detection of *Listeria*, bacteria were biotinylated prior to infection according to the manufacturers protocol. Pharmacological inhibitors were added 30 min prior to infection if not stated otherwise.

### Immunofluorescence microscopy and flow cytometric analysis

For intracellular Cxcl-2 or Cxcl-5 visualization, brefeldin A (0.5 µg/mL) was added to the cell culture medium 1 h after stimulation. Cells were fixed in 3% PFA and incubated with anti-Cxcl-2 or Cxcl-5 antiserum (1∶100). Nox4 was detected in formalin-fixed sections of mouse small intestine by incubation with a rabbit anti-Nox4 antiserum (1∶100) for 1 h at room temperature, followed after washing by a TR-conjugated secondary antibody. Cells were mounted in Vectashield Mounting Medium with Dapi (Vector Laboratories, Eching, Germany) and visualized using a Leica DM IRB Inverted Research Microscope with a TCS SP2 AOBS scan head (Leica Microsystems GmbH, Wetzlar, Germany). For fluorescent detection, immunolabelled *Listeria* was additionally stained with AF 555-conjugated secondary antibody prior to infection, or biotinylated bacteria were labelled by streptavidin-conjugated Cy3. For flow cytometry cells were trypsinized and fixed in Cytofix (BD Biosciences). Cxcl-2 or Cxcl-5 was stained following permeabilization in 0.5% saponin/1% FCS/PBS buffer. Analysis was performed on a FACS Calibur apparatus (BD Biosciences). The data acquisition on GFP***^+^*** (recorded in channel Fl-1) and Cxcl-2***^+^*** or Cxcl-5***^+^*** cells (Cy5-conjugated, Fl-4) was restricted to a total number of 10.000 events. The data acquisition on GFP***^+^*** (recorded in channel Fl-1) bacteria was restricted to a total number of 100.000 events. Flow cytometry cell sorting was performed using a MoFlo (XDP Upgrade, Beckman-Coulter) at the Cell Sorting Facility, Medical School, Hanover.

### Immunoblotting

Cell were lysed in 3∶1 WB/SB vol/vol (WB: 50 mM Tris, pH 7.4, 120 mM NaCl; SB: 250 mM Tris, pH 6.5, 8% SDS, 40% glycerol; supplemented with a proteinase inhibitor cocktail [Complete Mini, Roche Diagnostics]). Samples were sonified and the protein concentration was determined (DC Protein Assay; Bio-Rad Laboratories). Protein was separated on 11% acrylamide gels and blotted on nitrocellulose. Membranes were incubated overnight at 4°C with the primary antibody. Detection was performed using peroxidase-labelled goat anti–rabbit or goat anti-mouse secondary antibodies in combination with the ECL kit (GE Healthcare). Before restaining, membranes were stripped for 45 min at 50°C in 62.5 mM Tris HCl, pH 6.7, 100 mM ß-mercaptoethanol and 2% SDS, followed by three 15-min washing steps.

### PCR and quantitative RT-PCR analysis

Cells were divided after cell sorting. DNA extraction was performed following incubation in lysosyme (10 mg/mL), proteinase K (10 mg/mL), and 5% SDS using TRIzol (Invitrogen) according to the manufacturer's instruction. DNA was washed in sodium citrate (0.1 mM) and precipitated in 75% ethanol. *Listeria* genomic DNA was detected by PCR (*Taq* DNA polymerase from Invitrogen) using primers specific for the listerial *hly* gene (forward: 5′-ATG TAA ACT TCG GCG CAA CT-3′, reverse: 5′-TCG TGT GTG TTA AGC GGT TT-3′, annealing 57°C, cycles 35). A fragment encoding eukaryotic hypoxanthine phosphoribosyltransferase *(Hprt)* was amplified using oligonucleotides 5′-TGC TGA CCT GCT GGA TTA CA-3′ and 5′-GCT TAA CCA GGG AAA GCA AA-3′ (annealing temperature 59°C, cycles 32) as control. Amplification products were analysed on a 2% agarose gel and visualized with SYBR Safe (Invitrogen). Total RNA was extracted using the RNeasy Protect Cell Mini Kit (Qiagen) and first-strand cDNAs was synthesized using oligo-dT primers. Real-time PCR was prepared with absolute QPCR ROX mix (Thermoscientific), sample cDNA, intron-spanning forward and reverse primers, as well as the 6-carboxy-fluorescein-conjugated target probe provided in the commercial TaqMan gene expression assay for murine *Hprt1* and *Cxcl-2* or *Cxcl-5* (Applied Biosystems). Analysis were performed using an ABI PRISM Sequence Detection System 7000 (Applied Biosystems). Samples were normalized to the endogenous control. Results were calculated by use of the Δ2-CT method and are presented as fold induction of target gene transcripts in stimulated relative to unstimulated controls.

### Reactive oxygen radical detection

The fluorogenic probe 5-(and-6)-carboxy-2′,7′-dichlorofluorescein diacetate (DCF-DA, Invitrogen) was used for reactive oxygen intermediates (ROI) visualization. Prior to stimulation, cells were incubated with DCF-DA (10 µg/mL) for 30 min at 37°C. After stimulation or infection for 20 min cells were rapidly rinsed with PBS, fixed in 3% PFA, washed twice with PBS and analyzed by fluorescence microscopy. For quantitative analysis of oxygen radical production, cells were rinsed with PBS to remove the free probe, and lysed in 200µl of 1% Triton/H_2_O. The lysate was transferred into microcentrifuge tubes, sedimented at 8,000×g for 5 min at 4°C, and 100 µl aliquots were dispensed in 96-well plates in triplicate. The index of oxidation (DCF) was calculated as the ratio of fluorescence intensity as compared to an untreated control.

### Microinjection

Cells were grown in collagen-coated 8-well chamber slides (Nunc, Rochester, NY) continuously bathed in cell culture medium. The 70 kDa high molecular weight gap junction impermeant fluorescent compound Texas Red Dextran (Molecular Probes, 10 mg/mL) was mixed with either the <1 kDa low molecular weight Lucifer Yellow (Molecular Probes, 10 mg/mL) or 0.5 mM cumene hydroperoxide in injection buffer (25 mM HEPES, 125 mM K-acetate, 5 mM Mg-acetate, pH 7.1). Fluorescent mixtures were loaded into individual Femtotips II injection capillars (Eppendorf, Hamburg, Germany). Cells were transferred to a LSM 510 META laser scanning confocal microscope equipped with an inverted Axiovert 200M stand (Carl Zeiss, Germany) and single cell microinjection was performed by using an InjectMan NI2/Femtojet injector system at p_i_: 180 hPa, t_i_: 0.2s, p_c_: 25 hPa. A minimum of 10 microinjected cells were analyzed per experiment. To study gap junctional intercellular communication, cells were analysed by live imaging microscopy after 5 min incubation. For ROI donor cumene hydroperoxide stimulation, cells were incubated 1 h, washed, and incubated in prewarmed fresh cell culture medium for an additional 3 h in the presence of 0.5 µg/mL brefeldin A. Cells were fixed in 3% PFA and further analyzed by intracellular chemokine staining and fluorescence microscopy.

### Statistical analysis

All experiments were performed at least three times and results are given as the mean ± standard deviation (SD) of one representative experiment. Statistical analyses were performed using the Student's *t* test. A p value<0.05 (*) or <0.01 (**) was considered significant.

## Supporting Information

Figure S1 **(A)** m-IC_cl2_ cells were infected with wt (white square) or *hly* mutant (dark grey square) or *hly/plcA/plcB* triple mutant (light grey square) *Listeria monocytogenes*. The number of intracellular bacteria was determined after the indicated time by gentamycin-killing invasion assay. **(B)** Macrophage-like RAW 264.7 cells were infected at the indicated multiplicity of infection (m.o.i.) with viable (wt) or heat inactivated wt (h.i.), or *hly* mutant *L. monocytogenes*. The number of immunolabelled Cxcl-2***^+^*** cells was determined 4 h after infection by flow cytometry. All experiments were performed at a multiplicity of infection of 100∶1, if not stated otherwise. Results are representative for three independent experiments and are presented as mean ± SD (invasion) or show one representative experiment.
(1.3 MB TIF)

Figure S2 **(A)** m-IC_cl2_ cells were left uninfected (**Ai**, left) or infected with biotinylated (Cy3) wt *L. monocytogenes* (**Ai**, right). The number [%] of Cy3***^+^*** (*Listeria*-infected, blue) or immunolabelled Cxcl-2***^+^*** (red) cells was visualized 4 h after infection by flow cytometry. Single channel analysis (Cy3: FL-2; Cxcl-2: FL-4) was depicted on the side of the axis. **(Aii)** The number of Cy3***^+^*** (*Listeria*-infected) cells among activated, Cxcl-2***^+^*** cells and **(Aiii)** the proportion of Cxcl-2***^+^*** cells among Cy3***^+^*** (*Listeria*-infected) cells was demonstrated gating on the respective population. **(Aiv)** Flow cytometric analysis of *Listeria* by biotinylation and streptavidin-conjugated Cy3 labelling after growth in culture medium. **(Bi)** m-IC_cl2_ cells were infected with wt P*_actA_*-*gfp Listeria monocytogenes* at a multiplicity of infection of 10∶1. Intracellular *Listeria* (GFP***^+^***, green) and immunolabelled Cxcl-2 (red) was visualized 4 h after infection by fluorescence microscopy. Scale bar, 30 µm. Inserts at foci of infection (1–6) were enlarged (scale bar, 5 µm). Magnification ×100, counterstaining with Dapi (blue). (**Bii**–**Biii**) m-IC_cl2_ cells were left untreated or stimulated with lipopolysaccharide (LPS, 10 ng/mL). Cxcl-2 (**Bii**, red) or Cxcl-5 (**Biii**, red) was visualized 4 h after stimulation by fluorescence microscopy. Magnification ×400, counterstaining with Dapi (blue). Scale bar, 5 µm. **(C)** Illustration of the flow cytometric characterization of uninfected **(Ci)**, wt P*_actA_*-*gfp L. monocytogenes* infected **(Cii)** m-IC_cl2_ cells. Comparison of the gate setting **(Ciii)** used for flow cytometric cell sorting of GFP**^low^** (*Listeria*-negative) epithelial cells infected with *actA* mutant P*_sod_*-*gfp L. monocytogenes*. All infection experiments were performed at a multiplicity of infection of 100∶1, if not stated otherwise. Results are representative for three independent experiments and show one representative experiment.
(2.3 MB TIF)

Figure S3 **(A)** m-IC_cl2_ cells were infected with wt (white square) or *actA* mutant (green square) *Listeria monocytogenes*. The number of intracellular bacteria was determined after the indicated time by gentamycin-killing invasion assay. **(B)** m-IC_cl2_ cells were infected with *actA* mutant P*_actA_*-*gfp L. monocytogenes*. The ratio of immunolabelled Cxcl-2***^+^*** cells to GFP***^+^*** (*Listeria*-infected) cells was determined after the indicated time by flow cytometry. **(C)** Illustration of the expression of GFP in *actA* mutant *L. monocytogenes* carrying the *actA* P*_actA_*-*gfp* or the P*_sod_*-*gfp* reporter plasmid after growth in culture medium until mid-log phase. **(D)** Flow cytometric analysis of GFP expression by wt P*_actA_*-*gfp L. monocytogenes* after growth in culture medium **(Di)**, as compared to after isolation from infected intestinal epithelial cells **(Dii)**. Bacteria were isolated 4 h after infection, immunolabelled with anti-*Listeria* antibody, and analysed for GFP expression after gating. **(E)** m-IC_cl2_ cells were infected with wt (black) or *hly* mutant (white) P*_actA_*-*gfp L. monocytogenes*. The number of GFP***^+^*** (*Listeria*-infected) cells was determined after the indicated time by flow cytometry. Hemolytic **(F)** and cytolytic **(G)** activity of recombinant LLO (rLLO) as determined by hemoglobin release by red blood cells (RBC) or lactate dehydrogenase (LDH) release by m-IC_cl2_ cells, respectively. **(H)** Hemolytic activity in undiluted, sterile-filtered culture supernatant (bact free media, normalised for multiplicity of infection of 100∶1) of wt, *actA*, and *hly* mutant *L. monocytogenes* grown in m-IC_cl2_ cell culture medium. Hemoglobin release by red blood cells was measured by photometric spectroscopy. **(I)** m-IC_cl2_ cells were infected with wt, *actA*, and *hly* mutant *L. monocytogenes*. Cytolytic activity was measured by LDH release. In **(F–G)** DTT was used as solvent control, and in **(F–I)** hypotonic lysis in H_2_O was used as positive control. All infection experiments were performed at a multiplicity of infection of 100∶1. Results are representative for three independent experiments and are presented as mean ± SD (invasion, hemolysis, LDH) or show one representative experiment.
(7.3 MB TIF)

Figure S4 **(A)** Illustration of the bicistronic expression vector for simultaneous expression of the NF-κB subunit RelA/p65 and GFP. ires: internal ribosome entry site. **(B)** m-IC_cl2_ cells were transfected with an NF-κB-luciferase reporter in combination with an empty control vector (pFlag), a RelA/p65 expression plasmid [p(p65)], or the bicistronic p65-ires-gfp expression plasmid [p(p65)-ires-gfp]. Luciferase activity was determined 6 h after transfection in cell lysate by luminescence spectroscopy. **(C)** m-IC_cl2_ cells were transfected with an empty control vector (pFlag), a RelA/p65 expression plasmid [p(p65)], or the bicistronic p65-ires-gfp expression plasmid [p(p65)-ires-gfp]. Cxcl-2 was determined 26 h after transfection in cell culture supernatant by ELISA. **(D)** m-IC_cl2_ cells were transfected with an empty control vector (pFlag, left panel) or the bicistronic p65-ires-gfp expression plasmid [p(p65)-ires-gfp, right panel]. The number of GFP***^+^*** or immunolabelled Cxcl-2***^+^*** cells was visualized 26 h after transfection by flow cytometry. **(E)** m-IC_cl2_ cells were transfected with the bicistronic p65-ires-gfp expression plasmid [p(p65)-ires-gfp]. The number of GFP*^+^* (black square) or immunolabelled Cxcl-2***^+^*** (white diamond) cells was determined after the indicated time by flow cytometry. **(F)** m-IC_cl2_ cells were stimulated with LPS (10 ng/mL) in the absence or presence of brefeldin A (BFA, 0.5 µg/mL). The amount of Cxcl-2 secreted into the cell culture supernatant as well as found in the cell lysate was quantified by ELISA. **(G)** m-IC_cl2_ cells were infected with *actA* mutant P*_actA_*-*gfp L. monocytogenes* (white) or exposed to freshly prepared sterile cell culture supernatant from *Listeria* infected cells obtained at the indicated time points after infection (bact free supernatant, black). The number of immunolabelled Cxcl-2***^+^*** cells was determined 4 h after infection or stimulation by flow cytometry. **(H)** m-IC_cl2_ cells were infected with *actA* mutant P*_actA_*-*gfp L. monocytogenes*. The ratio of immunolabelled Cxcl-2***^+^*** cells to GFP***^+^*** (*Listeria*-infected) cells was determined 4 h after infection in the absence or presence of the indicated inhibitors by flow cytometry. Used inhibitors: thapsigargin (1 µM), CFTR II (1 µM), indomethacin (0.1 mM) or bumetanide (0.1 mM). All infection experiments were performed at a multiplicity of infection of 100∶1. Results are representative for three independent experiments and are presented as mean ± SD (luciferase, ELISA) or show one representative experiment.
(7.1 MB TIF)

Figure S5 **(A)** m-IC_cl2_ cells were left untreated (white) or stimulated by 10 ng/mL LPS (black) or 10 µM PMA (grey). Cxcl-2 was quantified 4 h after stimulation in cell culture supernatant by ELISA. **(B)** m-IC_cl2_ cells were infected with wt *Listeria monocytogenes* in the absence (black diamond) or presence of DPI (dark grey diamond, 0.1 mM) or UO-126 (light grey diamond, 10 µM). The number of intracellular bacteria was determined 4 h after infection by gentamycin-killing invasion assay. ns, not significant.
(1.2 MB TIF)

Figure S6 m-IC_cl2_ cells were treated with control small interfering RNA (siRNA) or *Nox4* siRNA and subsequently left untreated (white) or stimulated with 10 ng/mL LPS (black) or 10 µM PMA (grey). Cxcl-2 was determined 4 h after stimulation in cell culture supernatant by ELISA. ns, not significant.
(0.58 MB TIF)
