# Supplementary figures and images for: Potentiation of Epithelial Innate Host Responses by Intercellular Communication
Source: PLoS Pathog. 2010 Nov 18;6(11):e1001194. doi: 10.1371/journal.ppat.1001194 (PMC2987820; doi:10.1371/journal.ppat.1001194)

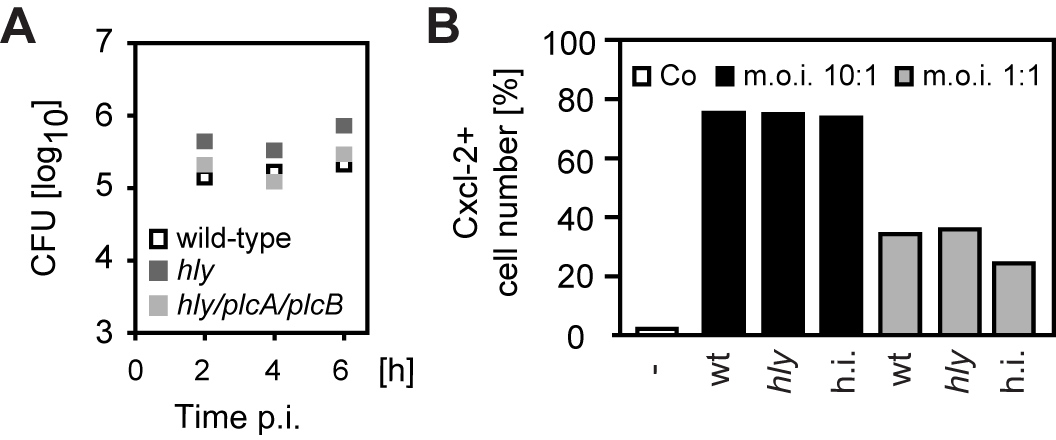

Supplement: Figure S1 — (A) m-ICcl2 cells were infected with wt (white square) or hly mutant (dark grey square) or hly/plcA/plcB triple mutant (light grey square) Listeria monocytogenes. The number of intracellular bacteria was determined after the indicated time by gentamycin-killing invasion assay. (B) Macrophage-like RAW 264.7 cells were infected at the indicated multiplicity of infection (m.o.i.) with viable (wt) or heat inactivated wt (h.i.), or hly mutant L. monocytogenes. The number of immunolabelled Cxcl-2+ cells was determined 4 h after infection by flow cytometry. All experiments were performed at a multiplicity of infection of 100∶1, if not stated otherwise. Results are representative for three independent experiments and are presented as mean ± SD (invasion) or show one representative experiment. (1.3 MB TIF) [file ppat.1001194.s001.tif]

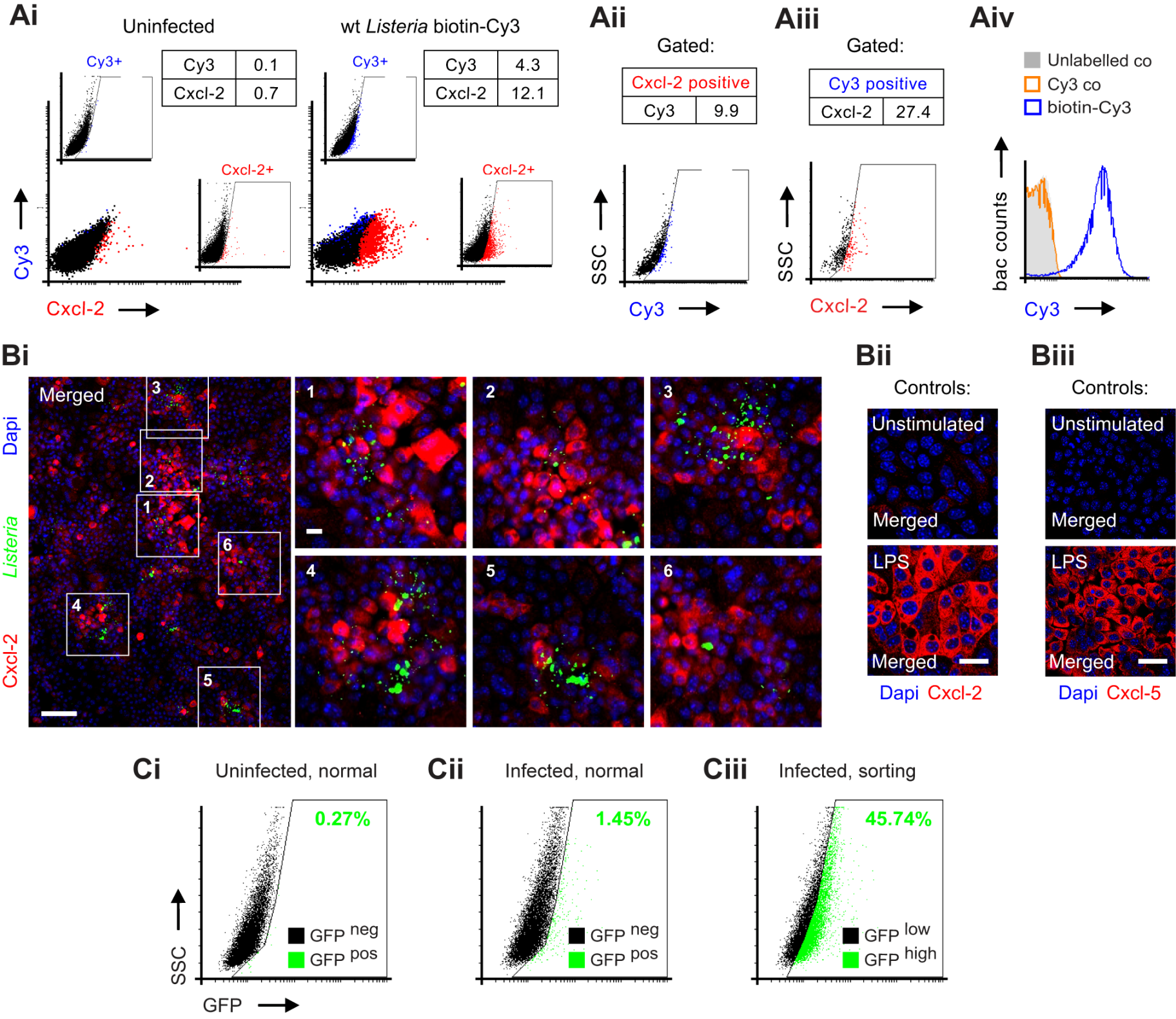

Supplement: Figure S2 — (A) m-ICcl2 cells were left uninfected (Ai, left) or infected with biotinylated (Cy3) wt L. monocytogenes (Ai, right). The number [%] of Cy3+ (Listeria-infected, blue) or immunolabelled Cxcl-2+ (red) cells was visualized 4 h after infection by flow cytometry. Single channel analysis (Cy3: FL-2; Cxcl-2: FL-4) was depicted on the side of the axis. (Aii) The number of Cy3+ (Listeria-infected) cells among activated, Cxcl-2+ cells and (Aiii) the proportion of Cxcl-2+ cells among Cy3+ (Listeria-infected) cells was demonstrated gating on the respective population. (Aiv) Flow cytometric analysis of Listeria by biotinylation and streptavidin-conjugated Cy3 labelling after growth in culture medium. (Bi) m-ICcl2 cells were infected with wt PactA-gfp Listeria monocytogenes at a multiplicity of infection of 10∶1. Intracellular Listeria (GFP+, green) and immunolabelled Cxcl-2 (red) was visualized 4 h after infection by fluorescence microscopy. Scale bar, 30 µm. Inserts at foci of infection (1–6) were enlarged (scale bar, 5 µm). Magnification ×100, counterstaining with Dapi (blue). (Bii–Biii) m-ICcl2 cells were left untreated or stimulated with lipopolysaccharide (LPS, 10 ng/mL). Cxcl-2 (Bii, red) or Cxcl-5 (Biii, red) was visualized 4 h after stimulation by fluorescence microscopy. Magnification ×400, counterstaining with Dapi (blue). Scale bar, 5 µm. (C) Illustration of the flow cytometric characterization of uninfected (Ci), wt PactA-gfp L. monocytogenes infected (Cii) m-ICcl2 cells. Comparison of the gate setting (Ciii) used for flow cytometric cell sorting of GFPlow (Listeria-negative) epithelial cells infected with actA mutant Psod-gfp L. monocytogenes. All infection experiments were performed at a multiplicity of infection of 100∶1, if not stated otherwise. Results are representative for three independent experiments and show one representative experiment. (2.3 MB TIF) [file ppat.1001194.s002.tif]

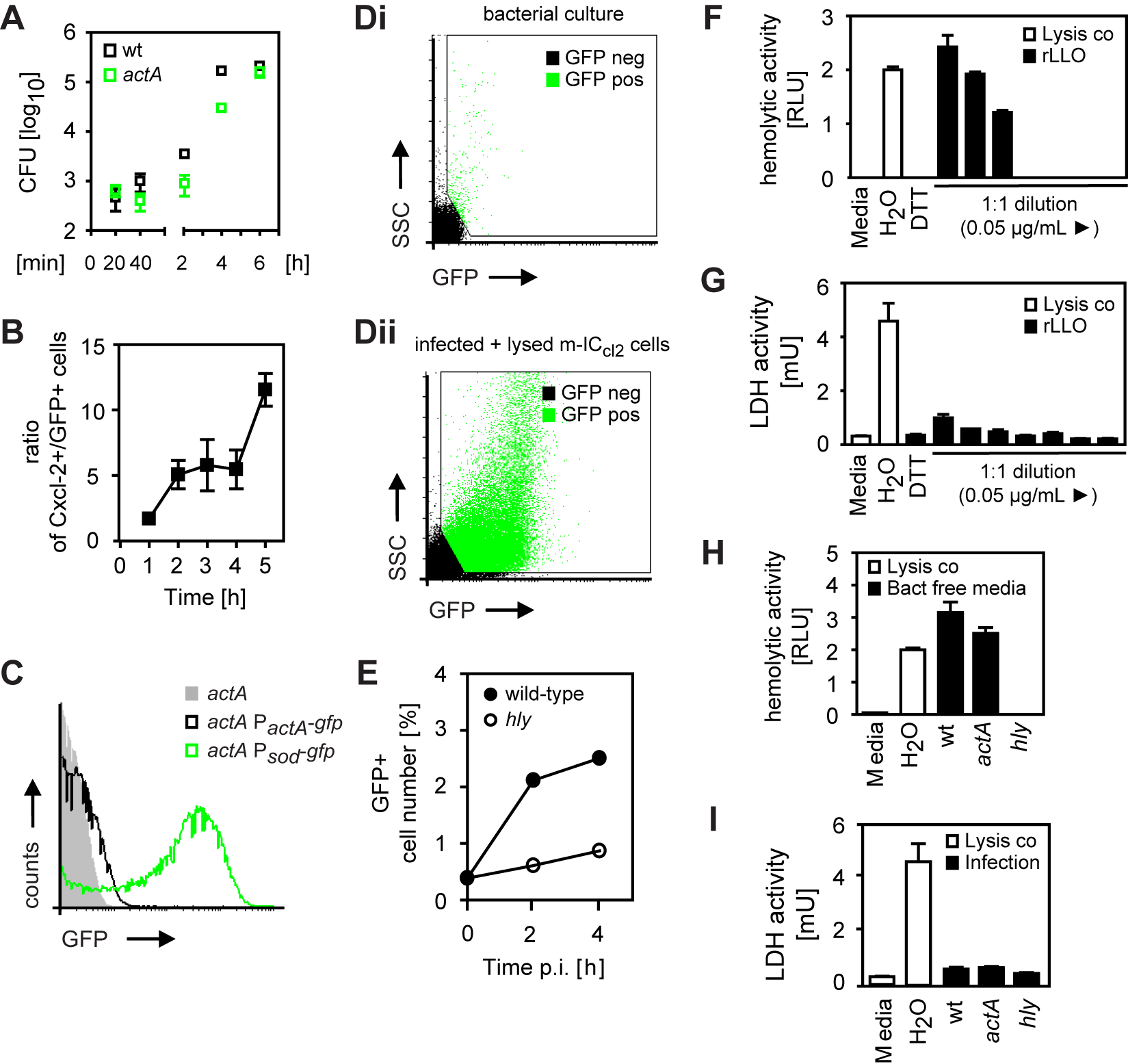

Supplement: Figure S3 — (A) m-ICcl2 cells were infected with wt (white square) or actA mutant (green square) Listeria monocytogenes. The number of intracellular bacteria was determined after the indicated time by gentamycin-killing invasion assay. (B) m-ICcl2 cells were infected with actA mutant PactA-gfp L. monocytogenes. The ratio of immunolabelled Cxcl-2+ cells to GFP+ (Listeria-infected) cells was determined after the indicated time by flow cytometry. (C) Illustration of the expression of GFP in actA mutant L. monocytogenes carrying the actA PactA-gfp or the Psod-gfp reporter plasmid after growth in culture medium until mid-log phase. (D) Flow cytometric analysis of GFP expression by wt PactA-gfp L. monocytogenes after growth in culture medium (Di), as compared to after isolation from infected intestinal epithelial cells (Dii). Bacteria were isolated 4 h after infection, immunolabelled with anti-Listeria antibody, and analysed for GFP expression after gating. (E) m-ICcl2 cells were infected with wt (black) or hly mutant (white) PactA-gfp L. monocytogenes. The number of GFP+ (Listeria-infected) cells was determined after the indicated time by flow cytometry. Hemolytic (F) and cytolytic (G) activity of recombinant LLO (rLLO) as determined by hemoglobin release by red blood cells (RBC) or lactate dehydrogenase (LDH) release by m-ICcl2 cells, respectively. (H) Hemolytic activity in undiluted, sterile-filtered culture supernatant (bact free media, normalised for multiplicity of infection of 100∶1) of wt, actA, and hly mutant L. monocytogenes grown in m-ICcl2 cell culture medium. Hemoglobin release by red blood cells was measured by photometric spectroscopy. (I) m-ICcl2 cells were infected with wt, actA, and hly mutant L. monocytogenes. Cytolytic activity was measured by LDH release. In (F–G) DTT was used as solvent control, and in (F–I) hypotonic lysis in H2O was used as positive control. All infection experiments were performed at a multiplicity of infection of 100∶1. Results are represent [file ppat.1001194.s003.tif]

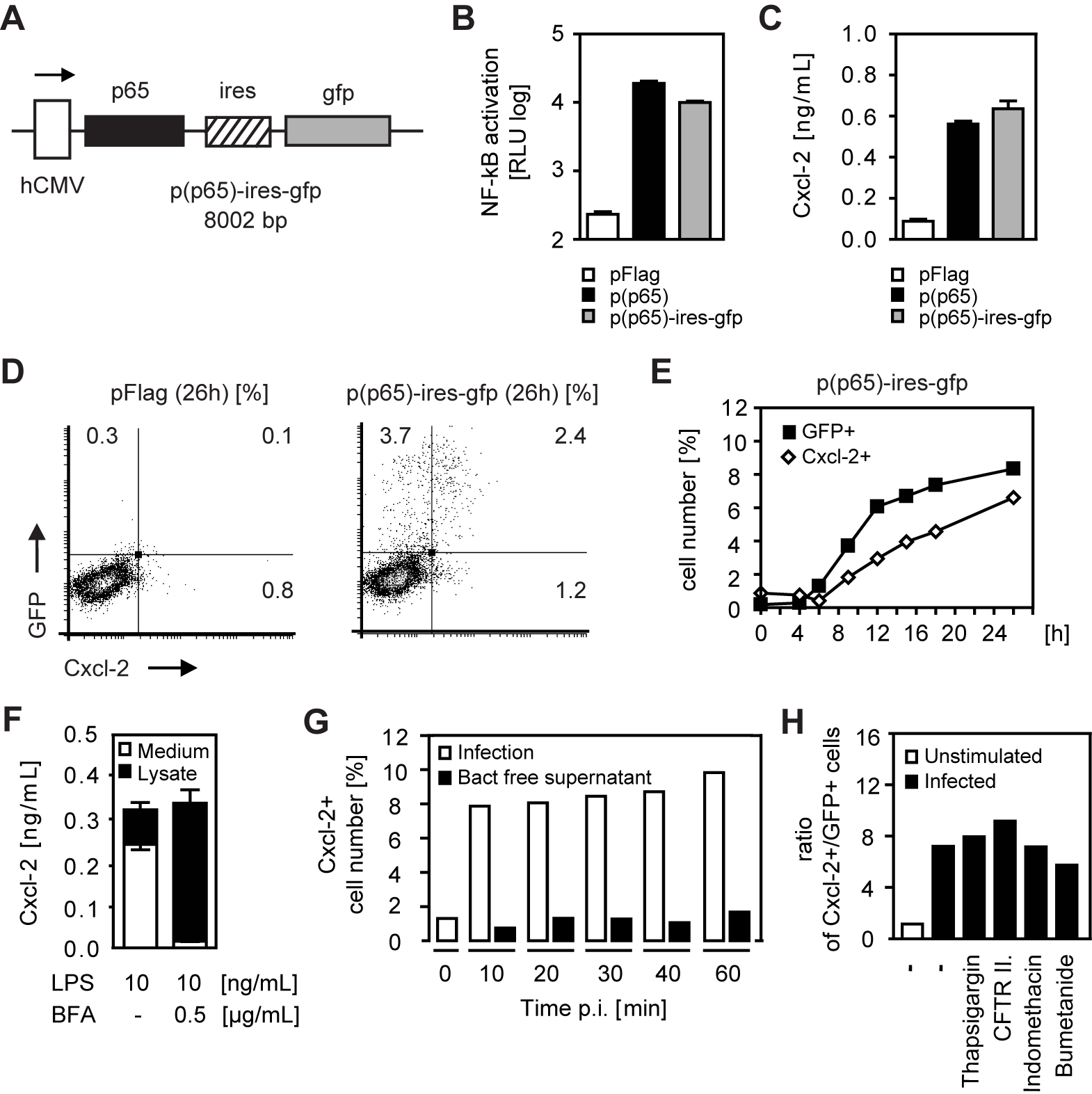

Supplement: Figure S4 — (A) Illustration of the bicistronic expression vector for simultaneous expression of the NF-κB subunit RelA/p65 and GFP. ires: internal ribosome entry site. (B) m-ICcl2 cells were transfected with an NF-κB-luciferase reporter in combination with an empty control vector (pFlag), a RelA/p65 expression plasmid [p(p65)], or the bicistronic p65-ires-gfp expression plasmid [p(p65)-ires-gfp]. Luciferase activity was determined 6 h after transfection in cell lysate by luminescence spectroscopy. (C) m-ICcl2 cells were transfected with an empty control vector (pFlag), a RelA/p65 expression plasmid [p(p65)], or the bicistronic p65-ires-gfp expression plasmid [p(p65)-ires-gfp]. Cxcl-2 was determined 26 h after transfection in cell culture supernatant by ELISA. (D) m-ICcl2 cells were transfected with an empty control vector (pFlag, left panel) or the bicistronic p65-ires-gfp expression plasmid [p(p65)-ires-gfp, right panel]. The number of GFP+ or immunolabelled Cxcl-2+ cells was visualized 26 h after transfection by flow cytometry. (E) m-ICcl2 cells were transfected with the bicistronic p65-ires-gfp expression plasmid [p(p65)-ires-gfp]. The number of GFP+ (black square) or immunolabelled Cxcl-2+ (white diamond) cells was determined after the indicated time by flow cytometry. (F) m-ICcl2 cells were stimulated with LPS (10 ng/mL) in the absence or presence of brefeldin A (BFA, 0.5 µg/mL). The amount of Cxcl-2 secreted into the cell culture supernatant as well as found in the cell lysate was quantified by ELISA. (G) m-ICcl2 cells were infected with actA mutant PactA-gfp L. monocytogenes (white) or exposed to freshly prepared sterile cell culture supernatant from Listeria infected cells obtained at the indicated time points after infection (bact free supernatant, black). The number of immunolabelled Cxcl-2+ cells was determined 4 h after infection or stimulation by flow cytometry. (H) m-ICcl2 cells were infected with actA mutant PactA-gfp L. monocytogenes. The ratio of immunolabelle [file ppat.1001194.s004.tif]

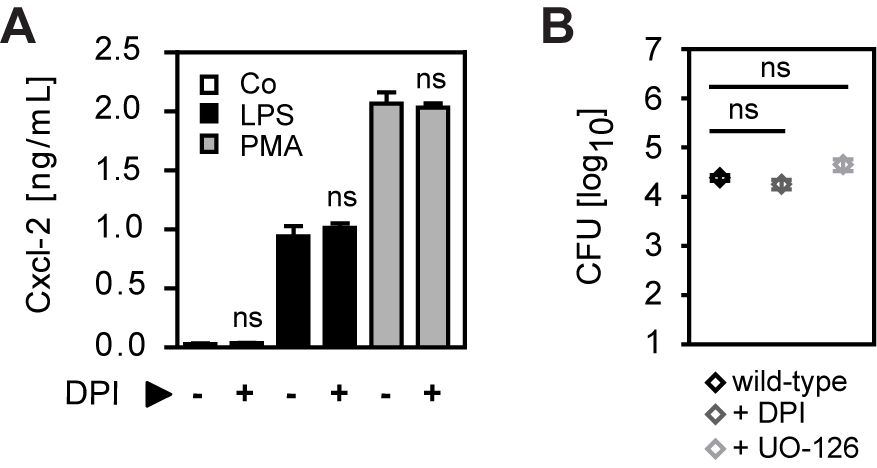

Supplement: Figure S5 — (A) m-ICcl2 cells were left untreated (white) or stimulated by 10 ng/mL LPS (black) or 10 µM PMA (grey). Cxcl-2 was quantified 4 h after stimulation in cell culture supernatant by ELISA. (B) m-ICcl2 cells were infected with wt Listeria monocytogenes in the absence (black diamond) or presence of DPI (dark grey diamond, 0.1 mM) or UO-126 (light grey diamond, 10 µM). The number of intracellular bacteria was determined 4 h after infection by gentamycin-killing invasion assay. ns, not significant. (1.2 MB TIF) [file ppat.1001194.s005.tif]

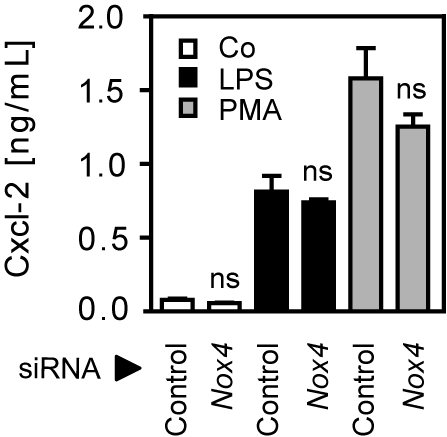

Supplement: Figure S6 — m-ICcl2 cells were treated with control small interfering RNA (siRNA) or Nox4 siRNA and subsequently left untreated (white) or stimulated with 10 ng/mL LPS (black) or 10 µM PMA (grey). Cxcl-2 was determined 4 h after stimulation in cell culture supernatant by ELISA. ns, not significant. (0.58 MB TIF) [file ppat.1001194.s006.tif]
